# Combined Thermodynamic-Kinetic Analysis of the Interfacial Reactions between Ni Metallization and Various Lead-Free Solders

**DOI:** 10.3390/ma2041796

**Published:** 2009-11-11

**Authors:** Tomi Laurila, Vesa Vuorinen

**Affiliations:** Electronics Integration and Reliability, Helsinki University of Technology, P.O.Box 3340, FIN-02015 TKK Espoo, Finland; E-Mail: vesa.vuorinen@tkk.fi (V.V.)

**Keywords:** phase diagram, diffusion, lead-free solders, thermodynamics, intermetallic compound layers, electroless Ni

## Abstract

In this paper we will demonstrate how a thermodynamic-kinetic method can be utilized to rationalize a wide range of interfacial phenomena between Sn-based lead-free solders and Ni metallizations. First, the effect of P on the interfacial reactions, and thus on the reliability, between Sn-based solders and electroless Ni/immersion Au (ENIG) metallizations, will be discussed. Next, the effect of small amounts of Cu in Sn-based solders on the intermetallic compound (IMC), which forms first on top of Ni metallization, will be covered. With the help of thermodynamic arguments a so called critical Cu concentration for the formation of (Cu,Ni)_6_Sn_5_ can be determined as a function of temperature. Then the important phenomenon of redeposition of (Au,Ni)Sn_4_ layer on top of Ni_3_Sn_4_ IMC will be discussed in detail. The reasons leading to this behaviour will be rationalized with the help of thermodynamic information and an explanation of why this phenomenon does not occur when an appropriate amount of Cu is present in the soldering system will be given. Finally, interfacial reaction issues related to low temperature Sn-Zn and Sn-Bi based solders and Ni metallization will be discussed.

## 1. Introduction

The manufacture of novel portable electronic devices with new functions and ever-higher performance is increasing concerns about the reliability of electronic products. Additional requirements are set by the implementation of lead-free materials, which demand careful consideration of compatibility between dissimilar materials for attaining high yield and reliability of lead-free equipment with an economically acceptable level of reliability testing. Therefore, especially under the mechanical shock loading, where the strain-rate hardening of the solder material forces cracks to propagate in the intermetallic layers, the role of interfacial intermetallic reactions as well as microstructural evolution in solder interconnections becomes more prominent.

Ni is often used as a diffusion barrier layer between Cu and Sn based solders in electronics, since the reaction rate of Ni with Sn is typically several orders of magnitude smaller than that of Cu [1]. This, in turn, results in thinner IMC layers in the Sn-Ni than in the Sn-Cu system. In addition, Ni/Au coatings provide desirable flat and uniform pad surfaces and they maintain good wettability, even after multiple reflows. Furthermore, Ni/Au coatings provide higher mechanical strength and resistance against thermal fatigue of lead-free solder interconnections than can be achieved when using organic solder preservatives (OSP) on Cu pads. Moreover, as an insulating material OSP has a disadvantage when connectors are assembled on the same board.

However, reliability problems have been reported when using Ni/Au coatings with Sn-based solders. Problems have appeared, especially when using the electroless Ni/immersion Au finish [2,3,4,5,6,7,8,9,10]. During the electroless coating process Ni is deposited on Cu together with phosphorous, because hypophosphate is used as a reducing agent in plating baths. It is the presence of this phosphorus in the surface finish layers that has been observed to be associated with the above-mentioned reliability problems. The root cause for the observed brittle fractures has been discussed in many papers dealing with the reactions between electroless Ni and SnPb-solders as well as lead-free solders, mainly near eutectic SnAgCu. However, the formation mechanism of the interfacial reaction products that causes the reliability problem has not yet been identified with any certainty.

When using Sn-Pb solder or pure Sn the Ni_3_Sn_4_ phase is typically the first phase to form in the reaction between Ni and Sn. However, when using Pb-free solders that include even small amounts of Cu the situation changes so that the first phase to form is (Cu,Ni)_6_Sn_5_ [1,11,12,13,14,15,16,17,18]. This Ni containing Cu_6_Sn_5_ intermetallic compound (IMC) is very brittle and has been observed to cause severe reliability problems, especially under mechanical shock loading [19]. If Pb-free solders do not contain enough Cu to enable the formation of (Cu,Ni)_6_Sn_5_, the Ni_3_Sn_4_ forms on top of Ni metallization [1,11]. Therefore, the determination of the critical Cu concentration, which causes the formation of (Cu,Ni)_6_Sn_5_ and its dependence on temperature and the presence of other alloying elements is of great importance.

Gold on top of the Ni layer provides adequate protection against the environment and ensures proper solderability. However, if the Au layer on top of the Ni metallization is too thick, other reliability problems will occur. This critical thickness is dependent on the temperature and time during operation and on the volume and composition of the solder used. The so-called redeposition of the AuSn_4_ IMC on top of the Ni_3_Sn_4_ layer has been frequently reported to take place [20,21,22,23,24]. This duplex IMC layer is brittle and thus has been observed to also cause reliability problems. In addition, when using low melting point solders, such as SnBi and especially Sn-Zn, the IMC layer structure at the interface may become highly complex [25,26,27].

In this review, the above-discussed phenomena described in the literature are rationalized with the help of a thermodynamic-kinetic approach. Details of the approach are given in [28,29]. The results from the literature are complemented with our own experimental results and calculations. Appropriate phase diagram and thermodynamic data, combined with the diffusion kinetic considerations provide an extremely useful tool to understand interfacial reaction issues. Root causes for the wide range of phenomena seen when using Ni metallization will be, at least in general terms, resolved. As a result, suggestions and guidelines to select materials, which are compatible with each other to be used in electronics fabrication, can be given.

## 2. Interfacial Reaction Issues with Ni(P) Metallization

During recent decades Ni/Au coatings have been extensively used in high-density component assemblies as a surface finish on printed wiring boards (PWB) because they have many advantages over other surface finishes such as hot air solder leveling (HASL) Sn finishes, as discussed above. Further, Ni/Au coatings provide higher mechanical strength, hardness and resistance against thermal fatigue of lead-free solder interconnections than can be achieved when using organic solder preservatives (OSP), Sn or Ag on Cu pads [30].

On the other hand, there are few typical reliability problems related to interfacial reactions between Ni/Au coatings with Sn based solders. Especially, the usage of electroless Ni/immersion Au (ENIG) finishes has lead into reduced reliability [2,3,4,5,6,7,8,9,10], because during the electroless plating process phosphorus is co-precipitated with Ni. It is the presence of phosphorus in the surface finish layers that has been observed to be associated with the above-mentioned reliability problems. Although the wetting occurs properly and the chemical reaction between Sn and Ni is evident, the interfacial strength is not adequate. The weakest interfacial reaction product readily fractures under mechanical stress and leaves behind an open circuit.

**Figure 1:**
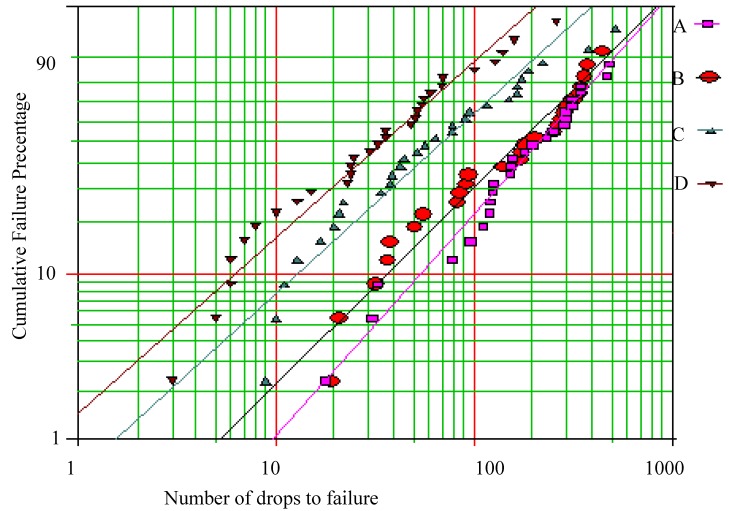
Weibull reliability plots from drop test results of assemblies with the Ni(P)|Au-finished soldering pads having the four different P- chemistries.

**Figure 2:**
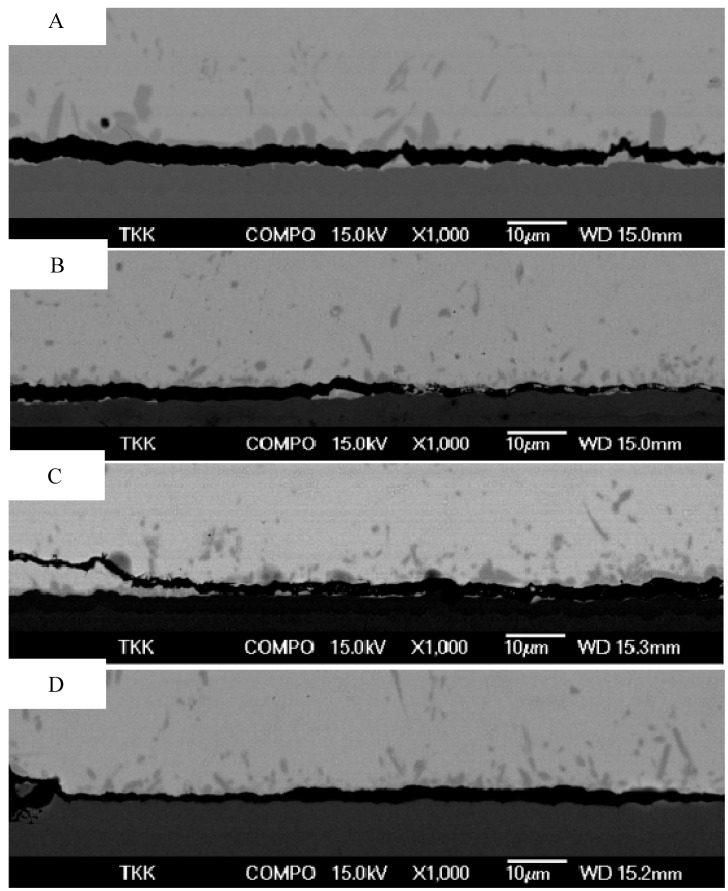
Failure mechanisms related to the different phosphorus chemistries.

Figure 1 shows the results from the drop test carried out according to the JESD22-B111 standard. The component was SnAgCu bumped (144 bumps having diameter 500 μm) CSP-component (12 mm × 12 mm), the solder was near eutectic SnAgCu and four different ENIG coatings on Cu pad were used. The phosphorus content of the coatings A and B was ~8 wt-%, coating C had over 10 wt-% phosphorus and D had less than 3 wt-% P The characteristic lifetimes (η) of the coatings were η_A_= 286, η_B_= 240, η_C_ = 101 and η_D_ = 50 drops to failure. As can be seen from Figure 2 the failure mechanisms related to the different phosphorus chemistries do not indicate detectable differences between the A, B and C. However, the low phosphorus coating (D) seems to be the weakest as no reaction products or solder can be seen between the crack and the coating.

The root cause for the brittle fracture has been discussed in many papers dealing with the reactions between electroless Ni and SnPb-solders as well as lead-free solders, mainly near eutectic SnAgCu [31,32,33,34,35,36,37,38,39,40,41]. However, the mechanism of formation of the interfacial reaction products that cause the reliability problems is not yet thoroughly understood. It has been proposed that the segregation of phosphorus on the PWB side of the fractured surface is responsible for the failure at the Ni_3_Sn_4_/Ni(P) interface in as-reflowed BGA (Ball Grid Array) solder interconnections [9]. Jang *et al*. [42] detected phosphorus enrichment in the Si/SiO_2_/Al/Ni(P)/63Sn37Pb multilayer structure after reflow and suggested that the mechanism of formation of the interfacial reaction layers is so-called solder assisted crystallization. This mechanism is based on the preferential dissolution of Ni from the Ni(P) layer, which leads to an increase in the phosphorus content of the upper part of the Ni(P) layer and the subsequent formation of Ni_3_P [42].

There are also suggestions that the brittle fracture is related to the redeposition of AuSn_4_ at the interface after high-temperature annealing [6,10,42,43,44,45,46]. However, this can only happen if the amount of Au (i.e., the thickness of the Au coating) exceeds its solubility in β-Sn at the annealing temperature [24]. In particular, when the intermetallic compound (IMC) formed at the interface after reflow is Cu_6_Sn_5_ or (Cu,Ni)_6_Sn_5_, (in the case of Cu-bearing solders), the latter explanation is not plausible as will be discussed later on.

**Figure 3:**
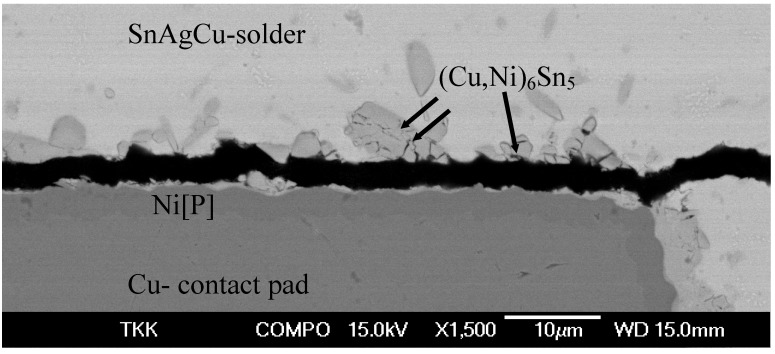
Brittle fracture (after drop test) in SnAgCu- solder / Ni[P] interface.

Figure 3 shows the interfacial microstructure between the NiP and near eutectic SnAgCu solder from the sample that has been under mechanical shock loading (drop test) after the assembly reflow. The point analysis taken from the interfacial IMC layer gives a composition of 40 at-% Cu, 15 at-% Ni and 45-at% Sn indicating that the IMC is (Cu,Ni)_6_Sn_5_, where Ni is dissolved into the Cu sublattice. Similar results have been reported in several papers dealing with the Sn-Cu-Ni system [1,11,12,13,14,15,16,17,18].

However, Hwang *et al*. have suggested that this phase could be Ni_3_Sn_2_, where Cu is dissolved into the Ni sublattice [47]. As both phases have the same NiAs structure with similar lattice parameters the analysis is difficult to confirm even with TEM. On the other hand, considering the relatively low solubility of Cu in (Ni,Cu)_3_Sn_2_ [47,48,49] it is more likely that the intermetallic layer is (Cu,Ni)_6_Sn_5_.

**Figure 4:**
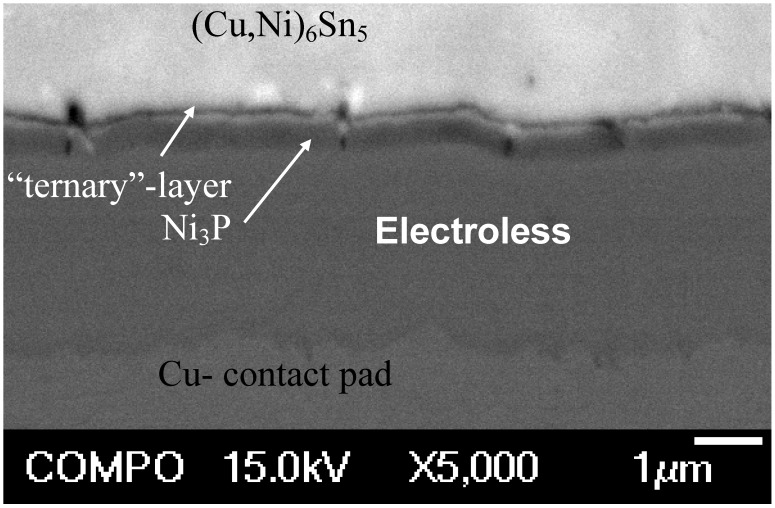
SEM micrograph revealing the presence of two reaction layers between the IMC and Ni[P] coating.

Between the IMC and Ni[P] coating two layers having different contrast can be observed (Figure 4). Next to Ni[P] there is a thin (less than 0.5 µm) dark layer and on the top of that a very thin layer with lighter contrast. Both layers form fast, since they are visible already after the soldering.

**Figure 5:**
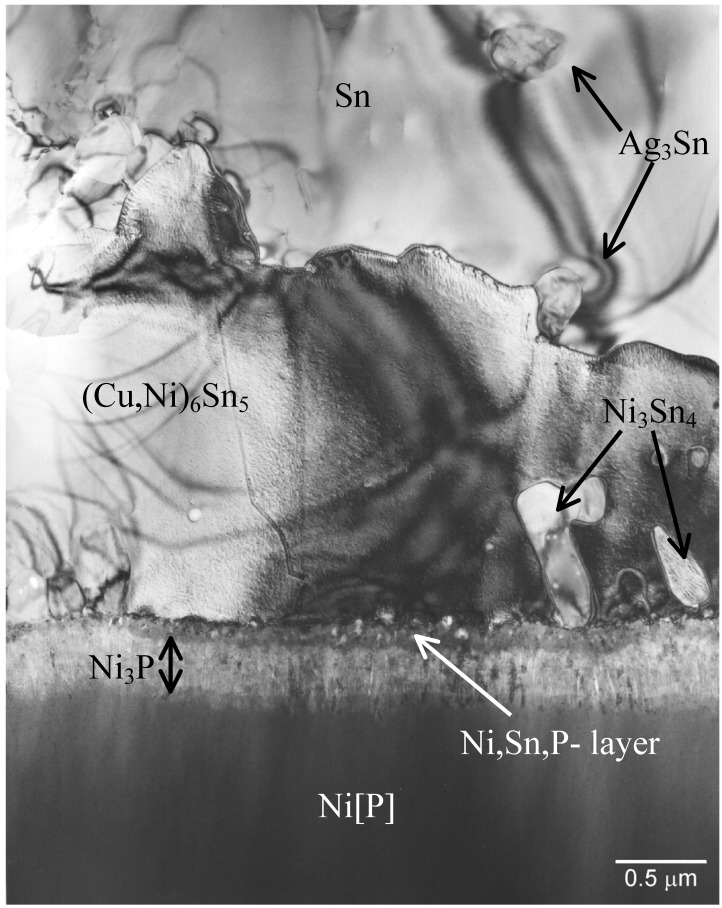
Bright-field TEM image of the interfacial region in the sample that have been reflowed five times.

Figure 5 taken from the sample that has been reflowed five times shows a bright-field TEM image from the IMC and phosphorus enriched area. From the TEM micrograph it is confirmed that the layer between the electroless Ni[P] and the (Cu,Ni)_6_Sn_5_ is not a single reaction layer but is composed of two layers, as also indicated by the SEM micrograph (Figure 4). The layer next to the electroless Ni[P] is a crystalline Ni_3_P. This phase was observed for the first time by Jang *et al*. when studying Si/SiO_2_/Al/Ni(P)/63Sn37Pb multilayer structure after the reflow [42].

**Figure 6:**
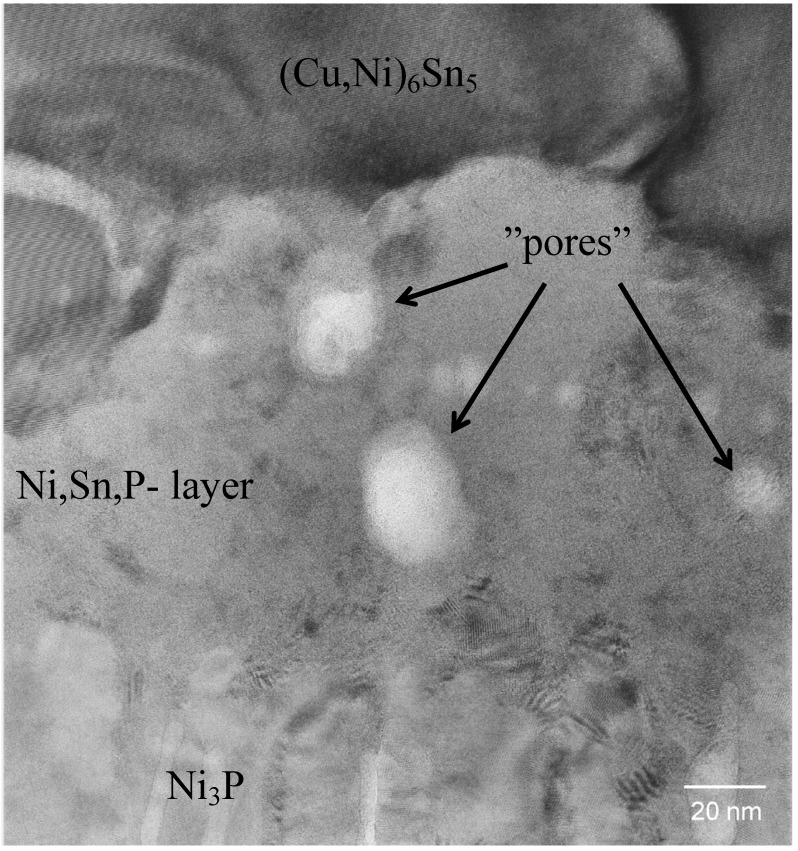
High-resolution phase contrast TEM image of the organic particles in the nanocrystalline Ni-Sn-P layer.

**Figure 7:**
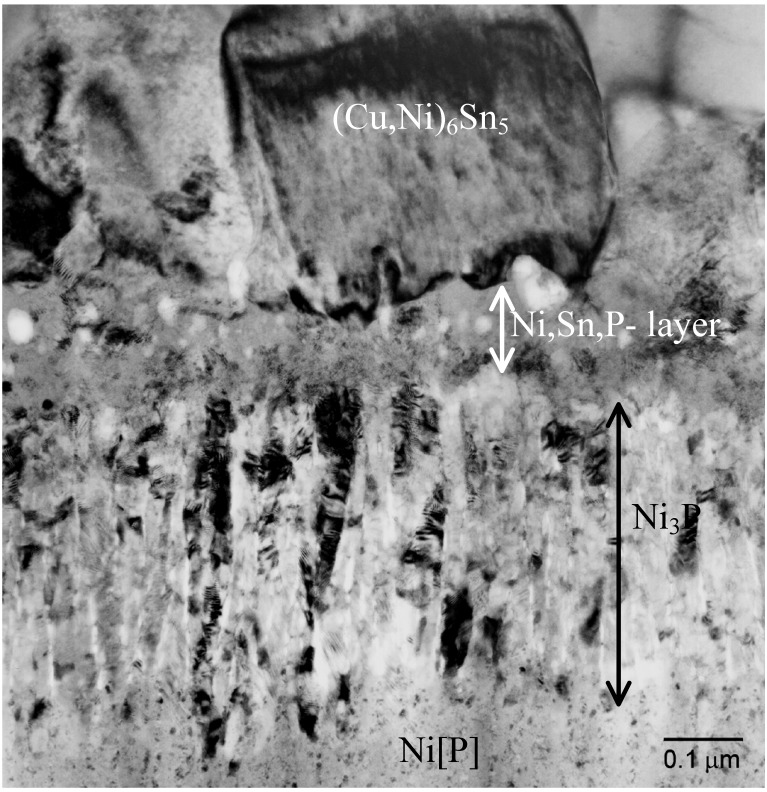
Bright field image from the reaction zone in the sample that has been annealed at 170 °C for 64 hours after the assembly reflow.

As shown in Figure 5 the crystalline Ni_3_P on top of the original Ni[P] coating has a columnar structure in which some organic impurities and tin seem to be concentrated between the columns. It is to be noted that there are few Ni_3_Sn_4_ crystals inside the (Cu,Ni)_6_Sn_5_ close to the ternary phase and some small Ag_3_Sn precipitates in Sn matrix (Figure 5). Figure 6 shows higher magnification bright-field TEM micrograph from the layer between (Cu,Ni)_6_Sn_5_ and Ni_3_P. The composition of this phase is estimated to be 55 at-% Ni, 35 at-% Sn and 10 at-% P and it has a nanocrystalline structure, based on the selected area diffraction (SAD) results. It is unlikely that this phase is Ni_2_SnP [50] or Ni_3_SnP [40,44] reported earlier. The ternary NiSnP layer contains numerous small defects that have a pore-like appearance as can be seen from Figure 5 and Figure 6. It is likely that these structural defects assists the crack propagation in the layer and thus makes interconnections behave in a brittle manner. The “pores” were also observed by Jeon *et al*. [51] in Ni[P] | (SnPb)_eut_ reactions and they claim that they are Kirkendall voids. It is difficult to accept that Kirkendall voids would form it the interfacial region in such a short time. Further, we did not observe any detectable change in the number or size of these “pores” in the ternary NiSnP layer when comparing samples that were reflow-soldered five times and annealed at 170 °C for 64 hours after the reflow (see Figure 7). In fact, Figure 6 suggests that the pores have internal structure and contain organic material that most probably originates from the Ni(P) plating bath.

When attempting to rationalize the formation of the observed reaction structures with the help of the solder-assisted crystallization approach we face several problems. The presence of a nanocrystalline NiSnP layer between the Ni_3_P and IMC cannot be explained by preferential dissolution of Ni from Ni[P] to liquid solder. This would require notable solid-state diffusion of Ni inside the Ni[P] coating so that Ni atoms can reach the Ni[P]/solder interface. This can hardly take place a short soldering process i.e., within less than a minute. Further, the solubility of P to liquid Sn (and Sn-based solders) is much larger than that of Ni [52]. As the solubility is directly related to dissolution rate of the given material, the dissolution rate of P to liquid Sn should consequently be larger than that of Ni. Therefore, the solder-assisted crystallization approach cannot be utilized for the more complex lead-free system considered here.

In order to explain the formation of above-mentioned reaction products more quantitatively the thermodynamic description of the quaternary Sn-Ni-P-Cu system is needed, for Ag can be ignored in the analysis, for the reasons explained earlier [1]. Unfortunately, there are not enough reliable data on the quaternary system as would be required for its critical thermodynamic assessment. However, it is still possible to rationalize the reaction sequence by making use of available binary Sn-P and Ni-P phase diagrams [53] as well as thermodynamic evaluations concerning the stability of ternary Sn-P-Ni liquids [54]. In addition, there exists a very recent experimental investigation on the Sn-P-Ni system, which also includes the determination of a new ternary phase [55]. However, these results were determined at high temperature (700 °C and above) with bulk materials, which are very different from the actual chemically deposited Ni(P) metallization, and represent global equilibrium conditions. As in soldering we are typically dealing with local (metastable) equilibria and the temperatures are much lower, the experimental results presented in [55] do not offer any help in the analysis.

The reaction between Ni(P) and liquid SnAgCu solder alloy starts with instant dissolution of a thin (flash) Au layer, which is followed by the dissolution of Ni and P. Due to the supersaturation of phosphorus in the Sn-rich solder a thin layer of new liquid (L2) is formed between the solid Ni(P) and the bulk liquid solder (L1). This argument is supported by the presence of a liquid miscibility gap in the binary Sn-P system that can extend to lower temperatures as a metastable miscibility gap also in the ternary Sn-P-Ni system [54]. Our preliminary calculations indicate that the metastable miscibility gap is strongly stabilized by dissolved Ni. When the two liquids become in local equilibrium with each other as well as with the Ni(P) substrate, Ni, P and Sn will redistribute between the liquids so that the liquid L2 contains large amounts of Ni and P and a small amount of Sn, while the Sn-rich liquid L1 has some Ni and a small amount of P. Because of the high P and Ni contents, the liquid L2 must be unstable at these low temperatures and therefore it turns rapidly to a nanocrystalline ternary layer providing a solid substrate for the (Cu,Ni)_6_Sn_5_ to nucleate and grow.

During cooling after the assembly reflow or during successive reflows the nanocrystalline NiSnP layer partly transforms into columnar Ni_3_P and the extra atoms, not being used in the formation of Ni_3_P, are rejected to the ternary NiSnP layer. In addition to P and Sn organic additives that are always present in electroless Ni(P) coatings also precipitate out at the interfaces between the columnar Ni_3_P crystals, as well as in the ternary NiSnP phase, where they are revealed as numerous small “pores” i.e., organic impurity particles, as shown in Figure 5 and Figure 6. Therefore, the Kirkendall voids reported earlier may, in fact, be organic particles. Also the white stripes between the columnar Ni_3_P crystals in Figure 5 as observed also by Matsuki, Ikuba, and Saka [50] seem to be organic material, because their contrast does not change by tilting the specimen.

**Figure 8:**
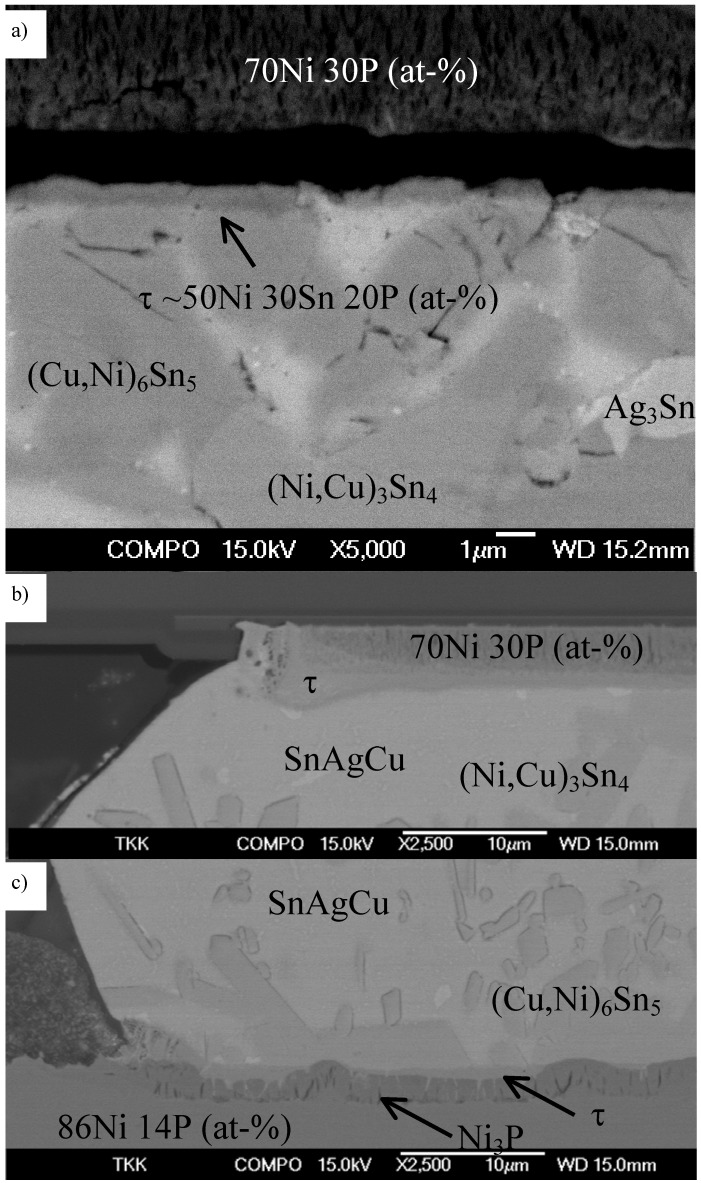
Backscattered SEM images (a) the component side, where the P concentration in Ni(P) is about 30 at-% annealed for 5 min at 250 °C, (b) the component side annealed for 20 min at 250 °C and, (c) the PWB side, where the P concentration in Ni(P) is about 14 at-%, annealed for 20 min at 250 °C.

It is interesting to find out that when the P content of the electroless Ni(P) coating is high enough, the formation of Ni_3_P is suppressed. This is shown in Figure 8a, where Ni(P) having high (30 at-%) phosphorus content has reacted with the near-eutectic Sn-Ag-Cu solder during reflow. Only the ternary NiSnP (τ) layer is visible between Ni(P) and the solder. In addition, the brittle fracture caused by mechanical shock loading has propagated at the interface between Ni[P] and the ternary layer. Furthermore, extensive spalling of two intermetallics (Cu,Ni)_6_Sn_5_ and (Ni,Cu)_3_Sn_4_, being in local equilibrium, can be seen. When the soldering time was extended to 20 minutes the difference between high (30 at-%) P content Ni[P] component under bump metallization and lower (14 at-%) P content Ni[P] PWB coating is even more evident (see Figure 8b and c). As the time above liquidus is now longer, more Ni (and P) is dissolved in the solder and the (Cu,Ni)_6_Sn_5_ near the component side interface is totally transformed to (Ni,Cu)_3_Sn_4_ as a result of the limited supply of Cu. It is also interesting to note from Figure 8 that (Ni,Cu)_3_Sn_4_ is never found in contact with the τ-phase. If (Cu,Ni)_6_Sn_5_ is present in the system, it is always located between the τ-phase and (Ni,Cu)_3_Sn_4_. When (Cu,Ni)_6_Sn_5_ is transformed into (Ni,Cu)_3_Sn_4_ (Figure 8b) the (Ni,Cu)_3_Sn_4_ phase is only found inside the bulk solder matrix away from the interface. This can be taken as an indication that (Ni,Cu)_3_Sn_4_ cannot exist in local equilibrium with the τ-phase whereas (Cu,Ni)_6_Sn_5_ can.

Based on the above experimental result it seems that the miscibility gap extends to the ternary systems in such a way that a higher P content in the original Ni(P) also results in a higher P content in the liquid L2 and therefore Ni_3_P can not form. This indicates that the higher phosphorus content will further stabilize the nanocrystalline NiSnP phase at the expense of the crystalline Ni_3_P, mainly for the following reason. It is known that the crystallization (and also the glass transition) temperature of an amorphous phase is usually lower at the eutectic point (19 at-% P in the Ni-P system) than near intermediate compounds or end-elements [56]. Therefore, we can expect that an increase in the P content of the nanocrystalline NiSnP layer will also increase its crystallization temperature, as the composition is shifted further away from the eutectic point. Further support for the effect of P on the stability of amorphous structures can be found from the literature, where it has been widely documented that when processing diffusion barriers for thin film applications amorphous structures are frequently realized by alloying elements such as B, C, N, Si, and P with transition metals [57].

## 3. Effect of Cu Concentration on the First IMC Formed on Top of Ni Metallization

**Figure 9:**
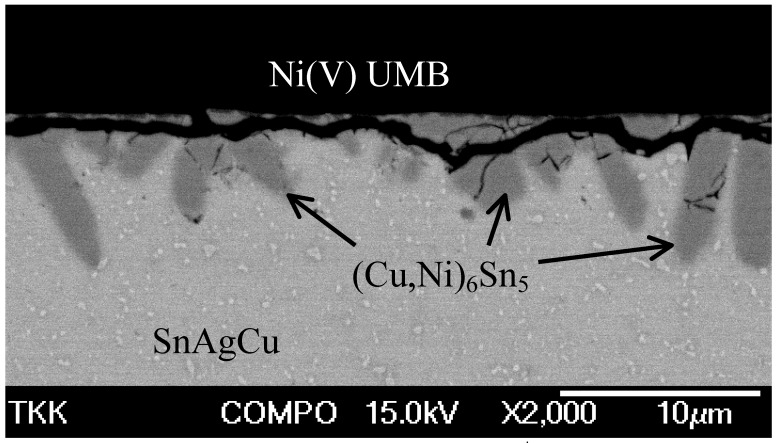
Cracking of the (Cu,Ni)_6_Sn_5_ reaction layer during drop testing.

When using liquid non-Cu-containing solders, such as SnPb, SnAg, or SnSb, the Ni_3_Sn_4_ phase is typically the first phase to form in the reaction between Ni and Sn. However, when using Pb-free solders that include small amounts of Cu the situation changes so that the first phase to form is (Cu,Ni)_6_Sn_5_. This Ni-containing Cu_6_Sn_5_ intermetallic compound (IMC) is very brittle and has been observed to cause severe reliability problems, especially under mechanical shock loading (see Figure 9).

On the other hand, if solders do not contain enough Cu to enable the formation of (Cu,Ni)_6_Sn_5_, the (Ni,Cu)_3_Sn_4_ forms on top of Ni metallization. Therefore, to determine the critical Cu concentration, which causes the formation of (Cu,Ni)_6_Sn_5_ and its dependence on temperature and the presence of other alloying elements is of great importance.

**Figure 10:**
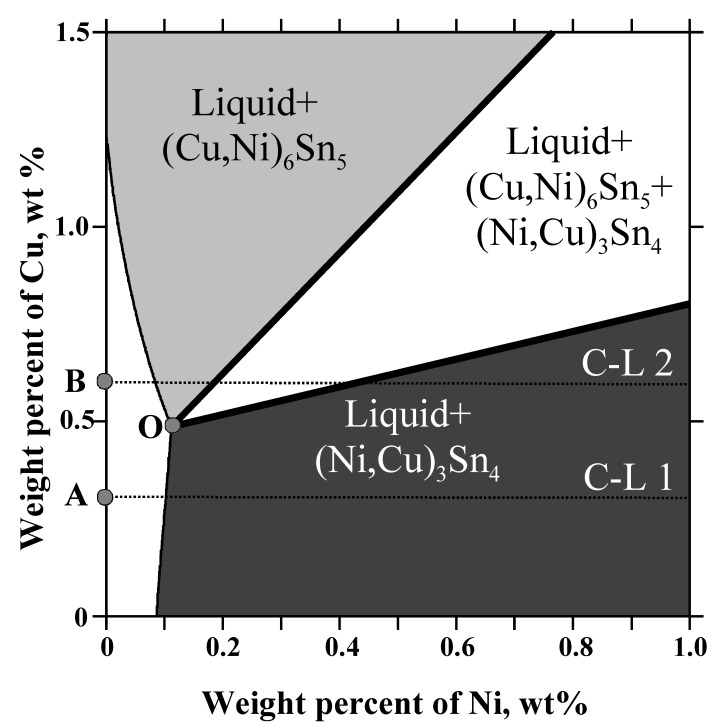
Tin-rich corner of the Sn-Cu-Ni phase diagram at 250 °C.

The thermodynamic-kinetic approach can be used for predicting the intermetallic reaction products in Cu-containing lead-free solders with Ni metallization. Figure 10 shows the Sn-rich corner of the isothermal section of the Sn-Cu-Ni phase diagram at 250 °C. If the Cu content of the solder is for example 0.3 wt-% (indicated with A in Figure 10) (Ni,Cu)_3_Sn_4_ nucleates at the Ni|solder interface because the dissolution of Ni into the solder changes the local nominal composition towards Ni along to the contact line 1 (C-L 1). Hence, in the cases when the local nominal composition (i.e., the average composition of solder near the interface) enters the dark grey area the liquid solder becomes in local equilibrium with (Ni,Cu)_3_Sn_4_. On the other hand, if the original Cu content of the solder is higher, say 0.6 wt-% (indicated with B in Figure 10), the dissolution of Ni (C-L 2) leads into local equilibrium between liquid and (Cu,Ni)_6_Sn_5_ (the light grey area in Figure 10). In addition, as the dissolution of Ni can continue until the metastable solubility (typically 2–3 times the stabile solubility) is reached, it is also possible that the local nominal composition enters the three phase triangle and then (Cu,Ni)_6_Sn_5_, Ni,Cu)_3_Sn_4_ and the liquid solder (with the composition marked with O) can become in local equilibrium, like seen in Figure 8a.

**Figure 11:**
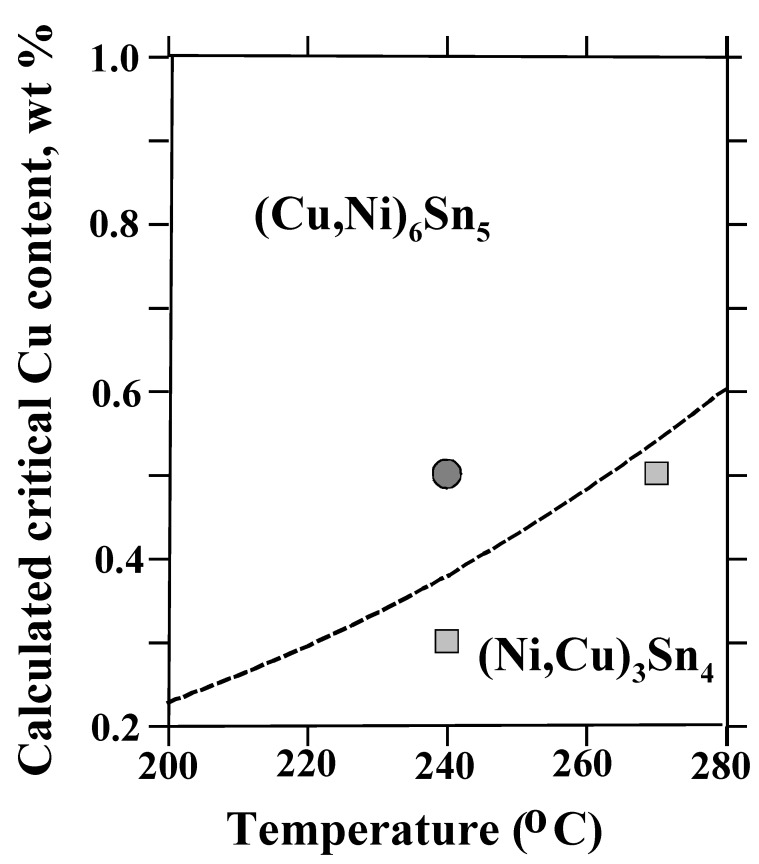
Calculated critical Cu content in liquid Sn to change interfacial reaction product from (Ni,Cu)_3_Sn_4_ to (Cu,Ni)_6_Sn_5_, as a function of temperature together with the experimental points.

**Figure 12:**
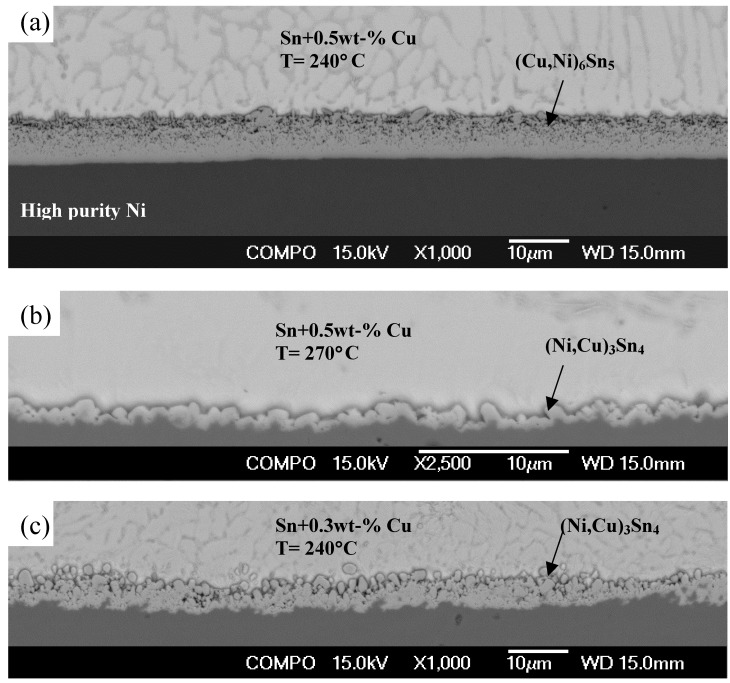
(a) Sn + 0.5wt-%Cu/Ni diffusion couple annealed at 240 °C, (b) Sn + 0.5wt-% Cu/Ni diffusion couple annealed at 270 °C, and (c) Sn+0.3wt-%Cu/Ni diffusion couple annealed at 240 °C for 60 min.

However, when the temperature is not constant (as in soldering) the phase equilibria will change as a function of temperature. These changes can be examined with the help of Figure 11, in which the apex of the three-phase triangle (point O in Figure 10), i.e., the critical Cu content of the solder, is presented as a function of temperature. Above (to the left of) the dotted line the primary intermetallic is (Cu,Ni)_6_Sn_5_ and below (to the right of) it the primary intermetallic is (Ni,Cu)_3_Sn_4_. The circles [(Cu,Ni)_6_Sn_5_] and square [(Ni,Cu)_3_Sn_4_] represent the experimental results shown in Figure 12. For example, when Cu content of the solder is 0.5 wt-% and reflow peak temperature is 240 °C (Cu,Ni)_6_Sn_5_ will form first, but when the temperature is increased up to 270 °C (Ni,Cu)_3_Sn_4_ becomes in local equilibrium with the solder, as seen from Figure 12a and b, respectively. The same change is also observed (see Figure 12 c) if the temperature is kept at 240 °C, but the Cu content of the solder is decreased to 0.3 wt-%. When comparing Figure 12a and b, it is interesting to note that the IMC thickness is clearly reduced when it is changed from (Cu,Ni)_6_Sn_5_ to (Ni,Cu)_3_Sn_4_, even when the temperature is increased. Therefore it can be concluded that the effect of temperature on the critical Cu composition is significant and thus it is possible to explain the (Cu,Ni)_6_Sn_5_ “precipitates” on the top of the (Ni,Cu)_3_Sn_4_ observed in [12,13,16,17] when the Cu content of the SnAgCu solder was 0.4 wt-%.

**Figure 13:**
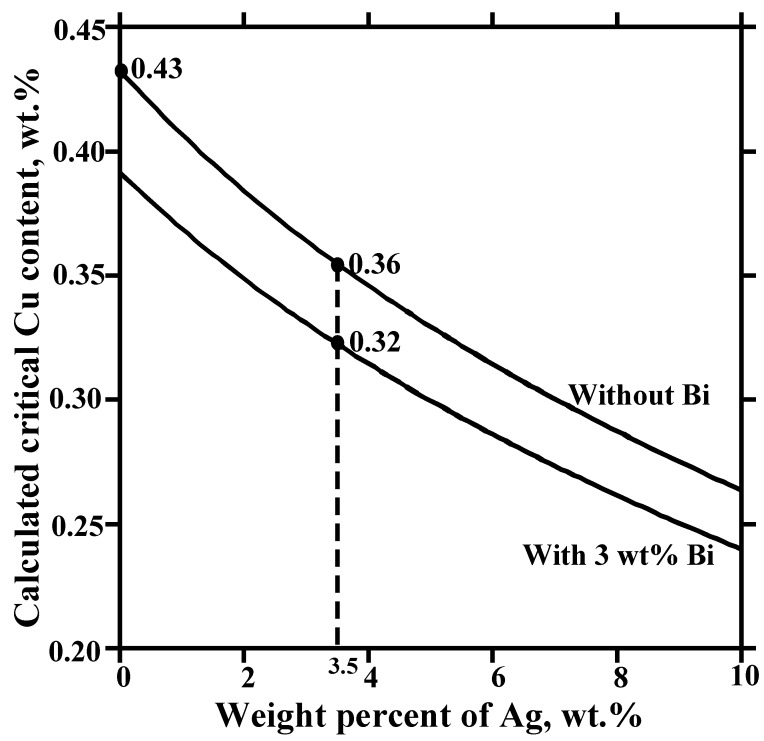
Effect of alloying Ag and Bi to Sn on the critical Cu content at T = 250 °C

The addition of other alloying elements will change the critical Cu content for (Cu,Ni)_6_Sn_5_ formation. Ho *et al*. [13] suggested that the existence of Ag in Sn-Ag-Cu solder does not have any effect, since it dissolves in neither (Ni,Cu)_3_Sn_4_ nor (Cu,Ni)_6_Sn_5_. This is not completely true, because the activities of all elements, not only in the compounds themselves but also in a liquid in (local) equilibrium with the compounds, determine the relative stabilities of (Ni,Cu)_3_Sn_4_ and (Cu,Ni)_6_Sn_5_. In order to investigate this, we have collected the thermodynamic interaction parameters in the liquid for the Sn-Cu-Ni-Ag system available in the literature and calculated the critical Cu content as a function of Ag content of the solder. As shown in Figure 13, the critical Cu content decreases when the Ag content of the solder is increased. Bismuth is another much-used alloying element in lead-free solders. If we add, say, 3 wt% Bi to the liquid alloy the critical composition of the liquid is further decreased. It means that both Ag and Bi will reduce the critical Cu content even though they do not dissolve in either of the compounds. An interesting subject for future studies is the effect of other alloying elements on the activities of components in both liquid and intermetallic compounds.

## 4. Redeposition of AuSn_4_ on Top of Ni_3_Sn_4_

Gold is generally used in electronics as a thin protective surface-finishing layer to ensure the solderability. Therefore, the amount of Au present in the soldering systems is usually quite small. However, the behavior of these small amounts of Au with other metals is theoretically interesting and is of great importance in soldering applications. One of the most important interactions is without a doubt the interplay between Ni and Au. About 10 years ago Mei *et al.* [9] revealed a problem that was peculiar to the Ni/Au metallization with SnPb solders. They discovered that after prolonged aging (150 °C for two weeks) the AuSn_4_ intermetallic compound, which had formed during the soldering in the bulk solder, redeposited at the solder/substrate interface. The reconstituted interface was significantly weakened and failed by brittle fracture along the surface between the redeposited AuSn_4_ and the Ni_3_Sn_4_ layer formed during the reflow.

Minor *et al.* [20] subsequently studied the mechanism of the redeposition of the AuSn_4_ intermetallic compound. They found out that the as-solidified solder interconnections contained dense distributions of small needle-like AuSn_4_ particles evenly distributed throughout the bulk solder. The interface between Ni and the bulk solder consisted of the layer of Ni_3_Sn_4_ that contained very small amount of Au. A coarse intermetallic layer developed above the Ni metallization during the aging. It thickened roughly as t^0.5^, i.e., indicating that the growth was diffusion controlled. The simultaneous depletion of AuSn_4_ needles from the bulk occurred. The redeposited intermetallic compound in the aged samples appeared to have composition close to AuSn_4_.

Ho *et al.* [21] investigated how the addition of small amounts of Cu into SnPb solder would influence the redeposition behavior of AuSn_4_. Again, after the reflow gold was completely absent from the interface and all the (Au_1-x_,Ni_x_)Sn_4_ intermetallic particles were evenly distributed throughout the bulk solder. At the Ni/solder interface Ni_3_Sn_4_ layer was formed except for the sample where solder included Cu. In this case the interfacial reaction layer was not Ni_3_Sn_4_ but Au-bearing (Cu_1-p-q_Au_p_Ni_q_)_6_Sn_5_ quaternary compound_._ The addition of 0.5 wt-% of Cu to SnPb solder completely inhibited the redeposition of (Au_1-x_,Ni_x_)Sn_4_. Only a layer of (Cu_1-p-q_Au_p_Ni_q_)_6_Sn_5_ was detected at the interface, and it actually reduced the consumption rate of Ni in the reaction.

Shiau *et al.* [22] carried out a study on reactions between lead-free Sn-Ag-Cu solder and Au/Ni finish in order to find out whether the redeposition of AuSn_4_ intermetallic compound would take place also in this lead-free system. Three solders were used: Sn3.5Ag, Sn4Ag0.5Cu and Sn3.5Ag0.75Cu. In the Sn3.5Ag alloy a thin layer of Ni_3_Sn_4_ had formed at the interface, while in the Sn3.5Ag0.75Cu a layer of (Cu_1-p-q_Au_p_Ni_q_)_6_Sn_5_ was formed after reflow. In the Sn4Ag0.5Cu solder both Ni_3_Sn_4_ and (Cu_1-p-q_Au_p_Ni_q_)_6_Sn_5_ were present near the interface. The formation of different IMC layers as a function of Cu concentration in the solder is readily rationalized with the concept of critical Cu concentration discussed in detail in Section 2 above. In the case of the Cu-free solder minor redeposition of (Au,Ni)Sn_4_ was observed at the interface. It should be noted that again in the systems with Cu no AuSn_4_ redeposition occurred.

**Figure 14:**
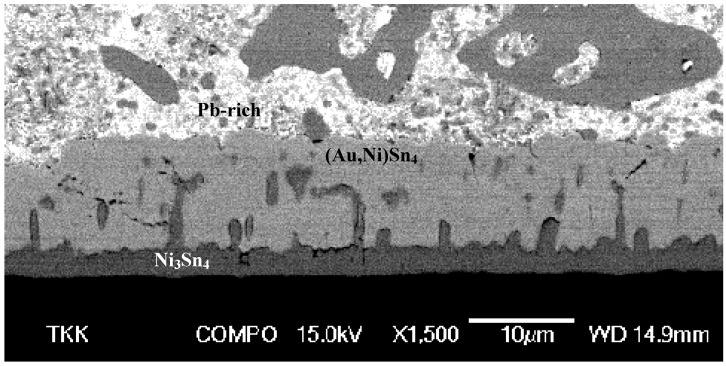
SEM micrograph from the (SnPbAg)/Ni/Au system after annealing for 250 hours at 150 °C.

Next we will present our own experimental results obtained with three different solders [Sn36.0Pb2.0Ag, Sn3.5Ag and Sn3.8Ag0.7Cu (wt-%)] and a 5 μm-thick Au layer on top of Ni metallization. Details of the experimental set-up and results can be found from [24]. After annealing for 250 h at 150 °C the (Au,Ni)Sn_4_ redeposition can be clearly seen to have taken place in the (SnPbAg)/Ni/Au system (Figure 14).

**Figure 15:**
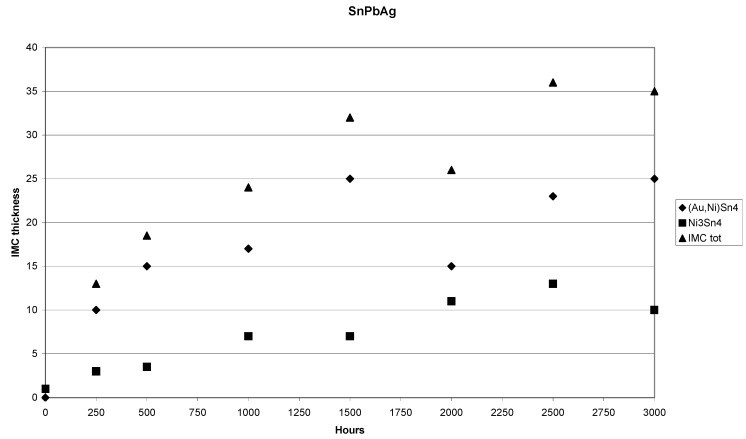
Plot of the IMC-thickness versus time in the (SnPbAg)/Ni/Au system.

The thickness of the (Au,Ni)Sn_4_ is remarkably large and also the numerous cracks visible in the layer indicate that it is most probably brittle. The same kinds of cracks have also been detected in another investigation [23]. Likewise, the interface between Ni_3_Sn_4_ and (Au,Ni)Sn_4_ shows some cracking (Figure 14). It should be emphasized that the excessive thickness of the (Au,Ni)Sn_4_ is related to the thick Au coating used in this study. Thus, the results of this study should not be compared directly to the earlier investigations where much thinner Au-layers were used [20,21,22,23]. Nevertheless, it is equally important to realize that the reaction mechanisms themselves are most likely identical. The formation of Pb-rich layer in front of the redeposited (Au,Ni)Sn_4_ intermetallic compound is also evident from the figure. The growth of the IMC layers with respect to time is shown in Figure 15. The corresponding minimum and maximum thickness values, compositions as well as the growth exponents determined by the regression analysis for the phases formed as a function of time are shown in Table 1.

The total IMC layer grows more or less with parabolic kinetics (n ≈ 0.5). As can be seen from Table 1 the growth of (Au,Ni)Sn_4_ is also diffusion controlled (the growth exponent n ≈ 0.5). The growth kinetics of Ni_3_Sn_4_ appears to be somewhat different as n ≈ 0.7. The thickness of the Ni_3_Sn_4_ layer also remains small and the IMC seems to have achieved its limiting value after 1,000 hours of annealing as the average thickness remains the same (after 1,500 hours) and the growth exponent seems to approach zero. (Figure 15 and Table 1).

**Table 1:** Minimum (d_min_), maximum(d_max_) and average thickness (d_av_) values, compositions as well as the growth exponents (n) determined by the regression analysis for the phases formed in (SnPbAg)/Ni/Au reaction couple.

Time@150°C [Hours] After reflow	(Ni_x_,Au_1-x_)_3_Sn_4_ thickness [µm] d< 1 µm	n	(Au_y_,Ni_1-y_)Sn_4_ thickness [µm] Not observed	n	IMC total thickness [µm] d~1 µm	n
250	d_av_ = 3 d_min_ = 2.5 d_max_ = 3.5 x = 1	0.7	d_av_ = 10 d_min_ = 6 d_max_ = 11 y = 0.5	0.5	d_av_ = 13 d_min_ = 9 d_max_ = 14	0.5
500	d_av_ = 3.5 d_min_ = 2.5 d_max_ = 4 x = 1	0.7	d_av_ = 15 d_min_ = 13 d_max_ = 18 y = 0.5	0.5	d_av_ = 19 d_min_ = 16 d_max_ = 22	0.5
1,000	d_av_ = 7 d_min_ = 6 d_max_ = 8 x = 1	0.7	d_av_ = 17 d_min_ = 14 d_max_ = 25 y = 0.5	0.5	d_av_ = 24 d_min_ = 20 d_max_ = 35	0.5
1,500	d_av_ = 7 d_min_ = 6 d_max_ = 8 x = 1	~ 0	d_av_ = 25 d_min_ = 23 d_max_ = 32 y = 0.5	0.5	d_av_ = 32 d_min_ = 30 d_max_ = 40	0.5
2,000	d_av_ = 11 d_min_ = 10 d_max_ = 12		d_av_ = 15 d_min_ = 13 d_max_ = 17		d_av_ = 26 d_min_ = 24 d_max_ = 27	
2,500	d_av_ = 13 d_min_ = 10 d_max_ = 14		d_av_ = 23 d_min_ = 20 d_max_ = 25		d_av_ = 36 d_min_ = 30 d_max_ = 40	
3,000	d_av_ = 10 d_min_ = 9 d_max_ = 12		d_av_ = 25 d_min_ = 19 d_max_ = 29		d_av_ = 35 d_min_ = 30 d_max_ = 40	

**Figure 16:**
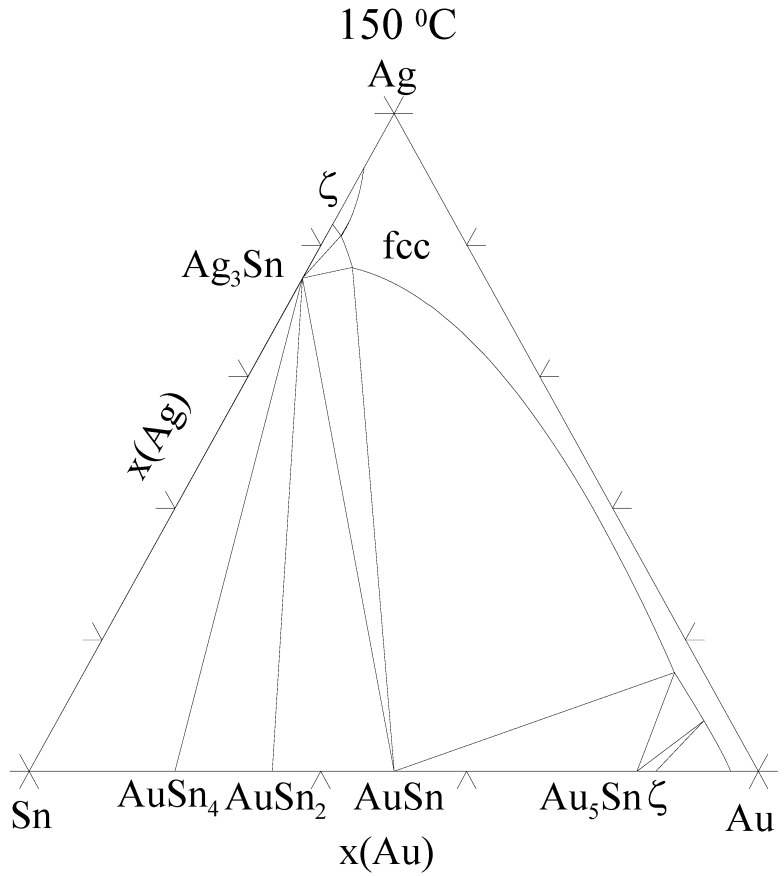
Isothermal section at 150 °C from the evaluated Ag-Au-Sn ternary phase diagram.

However, when the samples are further annealed the Ni_3_Sn_4_ continues to grow with more or less parabolic type kinetics and after 3,000 h the thickness of Ni_3_Sn_4_ is about 10 µm [that of (Au,Ni)Sn_4_ is about 25 µm]. The growth exponents for longer annealing times are not reported in the Table 1 as there is a notable drop in the thickness of the IMC’s after 2,000 h, at which point the growth seems to kick in again. This is most probably due to the limited experimental accuracy. The Ni content of the (Au,Ni)Sn_4_ was about 10 at-% [i.e., 50 % of Au atoms in the Au-sublattice have been replaced by Ni atoms (Table 1)]. In contrast, no Au could be detected inside Ni_3_Sn_4_ within the resolution limits of EDS. Likewise, no Ag was found in any of the above-mentioned IMC layers. It was detected only in the form of Ag_3_Sn compound throughout the solder matrix. Despite the fact that Ag_3_Sn can exist in local equilibrium with AuSn_4_ (Figure 16) at 150 °C it was not found at the interface. However, inside the bulk solder matrix Ag_3_Sn and AuSn_4_ precipitates were commonly found to be in contact.

**Figure 17:**
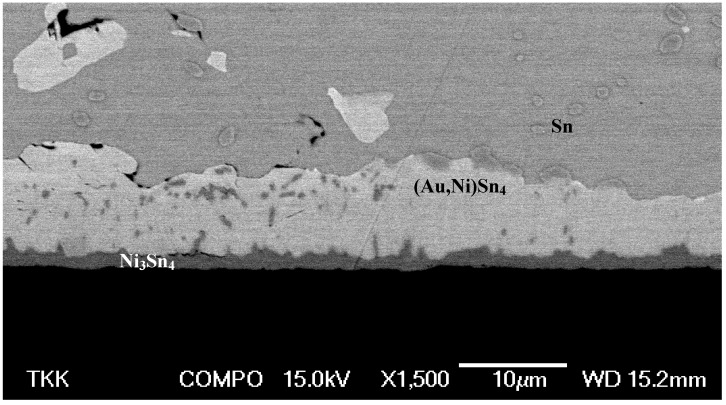
SEM micrograph from the (SnAg)/Ni/Au system after annealing for 250 hours at 150 °C.

**Figure 18:**
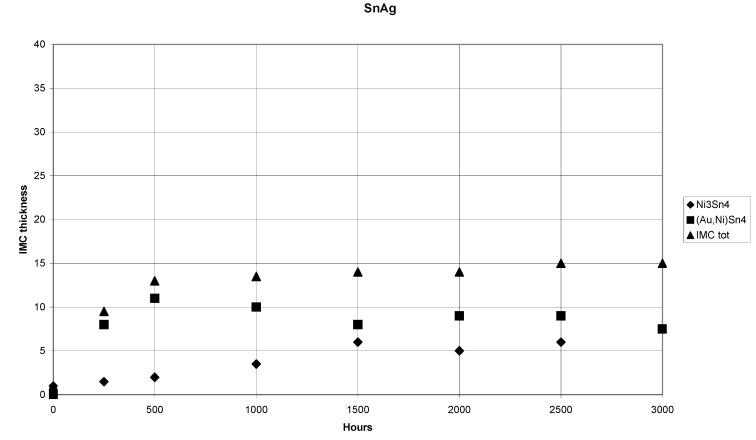
Plot of the IMC-thickness versus time in the (SnAg)/Ni/Au system.

The (SnAg)/Ni/Au system shows similar behavior as the (SnPbAg)/Ni/Au system. The interfacial structure after annealing at 150 °C for 250 hours is shown in Figure 17. The corresponding IMC thickness versus time is shown in Figure 18. Again the corresponding minimum/maximum thickness values, compositions as well as the growth exponents determined by the regression analysis for the phases formed as a function of time are shown in Table 2.

The total IMC layer grows with parabolic kinetics up to 500 hours (n ≈ 0.5). However, around 1,000 hours the growth exponent of the total IMC layer becomes zero and thus indicates that the limiting thickness of the layer has been reached. No further growth of total IMC layer thickness is observed up to 3,000 h of annealing. The growth kinetics of (Au,Ni)Sn_4_ is also parabolic (n ≈ 0.5) (Table 2). The thickness of the (Au,Ni)Sn_4_ is less than half of that in the (SnPbAg)/Ni/Au system and it seems that the limiting thickness is achieved around 500 hours. The time is the same when also the total IMC thickness ceases to increase. (Table 2). In fact after 1,000 hours of annealing the average thickness of the (Au,Ni)Sn_4_ phase starts to decrease and growth exponent becomes negative. This trend slowly continues when annealing times are extended to 3,000 hours. The growth of Ni_3_Sn_4_ seems to follow the linear kinetics whereas in the SnPbAg case the growth seemed to be a mixture of diffusion and reaction control.

**Table 2:** Minimum (d_min_), maximum (d_max_) and average thickness (d_av_) values, compositions as well as the growth exponents (n) determined by the regression analysis for the phases formed in (SnAg)/Ni/Au reaction couple.

Time@150 °C [Hours] After reflow	(Ni_x_,Au_1-x_)_3_Sn_4_ thickness d [µm] d~ 1µm	n	(Au_y_,Ni_1-y_)Sn_4_ thickness [µm] Not observed	n	IMC total thickness [µm] d~1.5 µm	n
250	d_av_ = 1.5 d_min_ = 1 d_max_ = 2 x = 1	1	d_av_ = 8 d_min_ = 5 d_max_ = 11 y = 0.5	0.5	d_av_ = 9 d_min_ = 7 d_max_ = 12	0.5
500	d_av_ = 2 d_min_ = 1.5 d_max_ = 2.5 x = 1	1	d_av_ = 11 d_min_ = 10򁅎 d_max_ = 12 y = 0.5	0.5	d_av_ = 13 d_min_ = 11 d_max_ = 14	0.5
1,000	d_av_ = 3.5 d_min_ = 2 d_max_ = 5 x = 1 (some ~6–8at% Cu detected)	> 1	d_av_ = 10 d_min_ = 6 d_max_ = 13 y = 0.5	< 0	d_av_ = 13,5 d_min_ = 10 d_max_ = 16	~ 0
1,500	d_av_ = 6 d_min_ = 5 d_max_ = 7 x = 1	> 1	d_av_ = 8 d_min_ = 6 d_max_ = 9 y = 0.5	< 0	d_av_ = 14 d_min_ = 11 d_max_ = 15	~ 0
2,000	d_av_ = 5 d_min_ = 4 d_max_ = 7 x = 1 (Ni layer consumed and (Cu,Ni,Au)_6_Sn_5_ Formed between the phases		d_av_ = 9 d_min_ = 8 d_max_ = 10 y = 0.5		d_av_ = 14 d_min_ = 12 d_max_ = 15	
2,500	d_av_ = 6 d_min_ = 4.5 d_max_ = 7.5 x = 1 (Ni layer consumed and (Cu,Ni,Au)_6_Sn_5_ Formed between the phases		d_av_ = 9 d_min_ = 5 d_max_ = 15 y = 0.5		d_av_ = 14 d_min_ = 11 d_max_ = 20	
3,000	d_av_ = 7.5 d_min_ = 6 d_max_ = 8 x = 1 (Ni layer consumed and (Cu,Ni,Au)_6_Sn_5_ Formed between the phases		d_av_ = 7.5 d_min_ = 10 d_max_ = 13 y = 0.5		d_av_ = 15 d_min_ = 13 d_max_ = 20	

**Figure 19:**
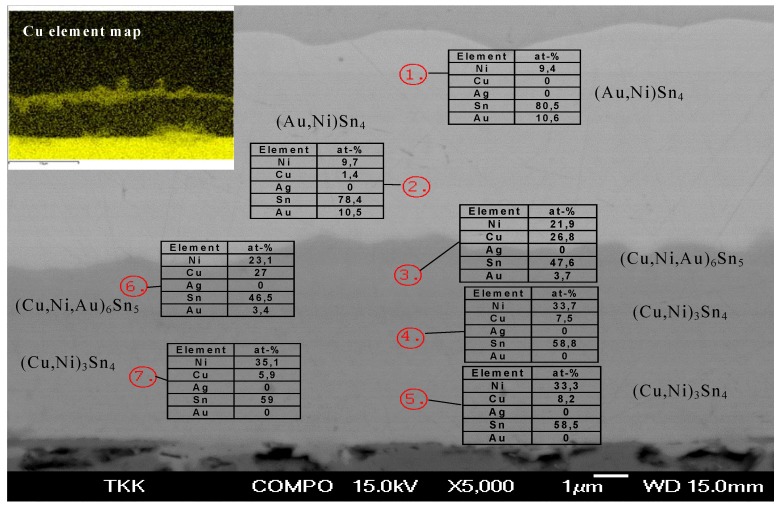
SEM micrograph together with point analyses and Cu element map from the (SnAg)/Ni/Au system after annealing for 2,000 hours at 150 °C.

It should be noted that the average thickness of the (Ni,Au)_3_Sn_4_ continues to increase after 1,000 hours, whereas that of (Au,Ni)Sn_4_ decreases and the growth exponents exceeds 1. This indicates that the Ni_3_Sn_4_ layer is partly growing at the expense of the (Au,Ni)Sn_4_ layer. The Ni content of the (Au,Ni)Sn_4_ is again around 10 at-%. On the contrary, no Au can be detected inside Ni_3_Sn_4_ (Table 2). After 2,000 hours the Ni layer seems to be totally consumed and (Cu,Ni,Au)_6_Sn_5_ is formed between (Au,Ni)Sn_4_ and (Ni,Cu)_3_Sn_4_ indicating that the underlying Cu starts to participate in the reactions. This (Cu,Ni,Au)_6_Sn_5_ layer continues to grow slowly between the (Au,Ni)Sn_4_ and (Ni,Cu)_3_Sn_4_ phases as shown in Figure 19. Because Cu starts to take part in the reactions, the growth exponents for the longer annealing times could not be determined.

**Figure 20:**
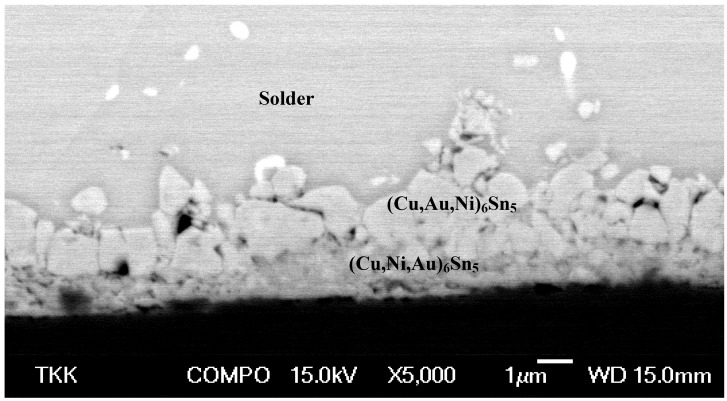
SEM micrograph from the (SnAgCu)/Ni/Au system after annealing for 250 hours at 150 °C.

In comparison to the (SnPbAg)/Ni/Au and (SnAg)/Ni/Au systems, the (SnAgCu)/Ni/Au system behaves quite differently. From Figure 20 it can be seen that no redeposition of AuSn_4_ intermetallic compound takes place after annealing at 150 °C for 250 hours (or up to 1,500 hours). The morphology of the structure shown in the micrograph (Figure 20) and the compositional analyses indicated that there are two reaction product layers with different Au to Ni ratios. The top layer is (Cu,Au,Ni)_6_Sn_5_, where the Au content is about 12 at-%, and the Ni content is about 2 to 3 atomic percents.

**Figure 21:**
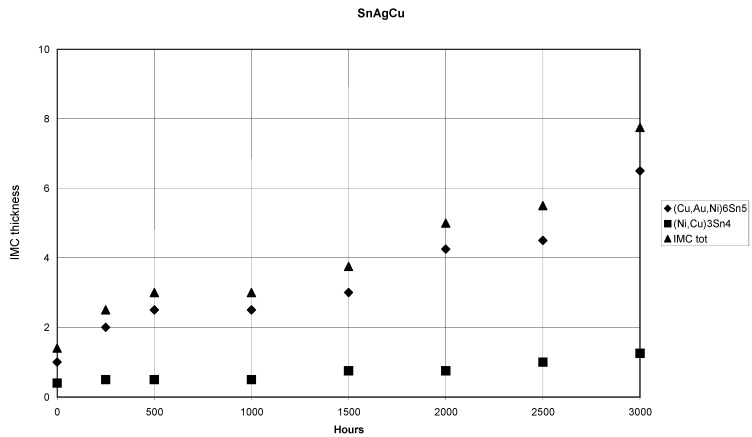
Plot of the IMC-thickness versus time in the (SnAgCu)/Ni/Au system.

The lower (Cu,Ni,Au)_6_Sn_5_ layer contains only about 5 at-% Au and about 17 at-% Ni. Some of the Ni signal may arrive from the underlying Ni substrate owing to the small thickness of the lower reaction layer. The total IMC thickness (Figure 21) is also small when compared to the (SnAg)/Ni/Au and (SnPbAg)/Ni/Au systems (Figure 15 and Figure 18) achieving about 7–8 µm after 3,000 hours of annealing. It is to be noticed that the y-axis in Figure 21 has different scale than those in Figure 15 and Figure 18. The corresponding minimum/maximum thickness values and compositions for the phases formed as a function of time are shown in Table 3. As the thickness increase of the IMC’s in this system after 250 hours of annealing was almost negligible (Table 3) we could not determine the growth exponents for this case. Based on the results obtained, it seems that the growth of the Cu_6_Sn_5_ is restricted by the limited supply of Cu in the solder. This is also the most probable explanation for the experimental observations made by Ho *et.al.* [21].

The first and foremost difference between the three systems investigated is that in both (SnAgPb)/Ni/Au and (SnAg)/Ni/Au systems the first phase to form is Ni_3_Sn_4_ whereas in the (SnAgCu)/Ni/Au system the first phase is (Cu,Ni,Au)_6_Sn_5_. Since this is strongly related to the differences observed in the redeposition behaviour of AuSn_4_ as (Au,Ni)Sn_4_ it is important to know why the formation of (Cu,Ni,Au)_6_Sn_5_ takes place instead of Ni_3_Sn_4_ in the (SnAgCu)/Ni/Au system. This issue was addressed in detail above and thus is not repeated here.

In order to analyze the reasons for the redeposition of the AuSn_4_ all the appropriate interfaces formed in the reaction couples i.e., Ni_3_Sn_4_/AuSn_4,_ Cu_6_Sn_5_/AuSn_4_ and AuSn_4_/solder, must be investigated to check that they fulfill the requirement of local equilibrium with each other. The (Au,Ni)Sn_4_/solder interface will be investigated first.

**Table 3:** Minimum (**d_min_**), maximum (**d_max_**) and average thickness (**d_av_**) values and compositions for the phases formed in (SnAgCu)/Ni/Au reaction couple.

Time@150°C [Hours] After reflow	(Cu_1-p-q_,Au_p_,Ni_q_)_6_Sn_5_ thickness [µm] d~ 1 µm	(Cu_1-r-s,_Ni_r_,Au_s_)_6_Sn_5_ thickness [µm]	IMC 2 thickness [µm]	IMC total thickness [µm] d~1 µm
250	d_av_ = 2 d_min_ = 1.5 d_max_ = 4 p = 0.25 and q < 0.1	d< 1 r = 0.3 and s < 0.1		d_av_ = 2.5 d_min_ = 2 d_max_ = 5
500	d_av_ = 2.5 d_min_ = 2 d_max_ = 4 p = 0.25 and q < 0.1	d< 1 r = 0.3 and s < 0.1		d_av_ = 3 d_min_ = 2.5 d_max_ = 5
1,000	d_av_ = 2.5 d_min_ = 2 d_max_ = 4 p = 0.25 and q < 0.1		d< 1 Ni_11_Cu_35_Au_7_Sn_47_	d_av_ = 3 d_min_ = 2.5 d_max_ = 5
1,500	d_av_ = 3 d_min_ = 2.5 d_max_ = 4 p = 0.25 and q < 0.1		d< 1 Ni_23_Cu_30_Au_5_Sn_42_	d_av_ = 3.5 d_min_ = 2.5 d_max_ = 5
2,000	d_av_ = 4.25 d_min_ = 3.5 d_max_ = 5 p = 0.20 and q = 0.2		d_av_ = 0.75 d_min_ = 0.5 d_max_ = 1 x ~ 0.5 and y < 0.1	d_av_ = 5 d_min_ = 4 d_max_ = 5.5
2,500	d_av_ = 4.5 d_min_ = 3.5 d_max_ = 5 p = 0.20 and q = 0.2		d_av_ = 1 d_min_ = 1 d_max_ = 1.5 x ~ 0.5 and y < 0.1	d_av_ = 5.5 d_min_ = 4.5 d_max_ = 6
3,000	d_av_ = 6.5 d_min_ = 6 d_max_ = 7 p = 0.20 and q = 0.2		d_av_ = 1,25 d_min_ = 1 d_max_ = 1,5 x ~ 0.5 and y < 0.1	d_av_ = 7.75 d_min_ = 7 d_max_ = 8

**Figure 22:**
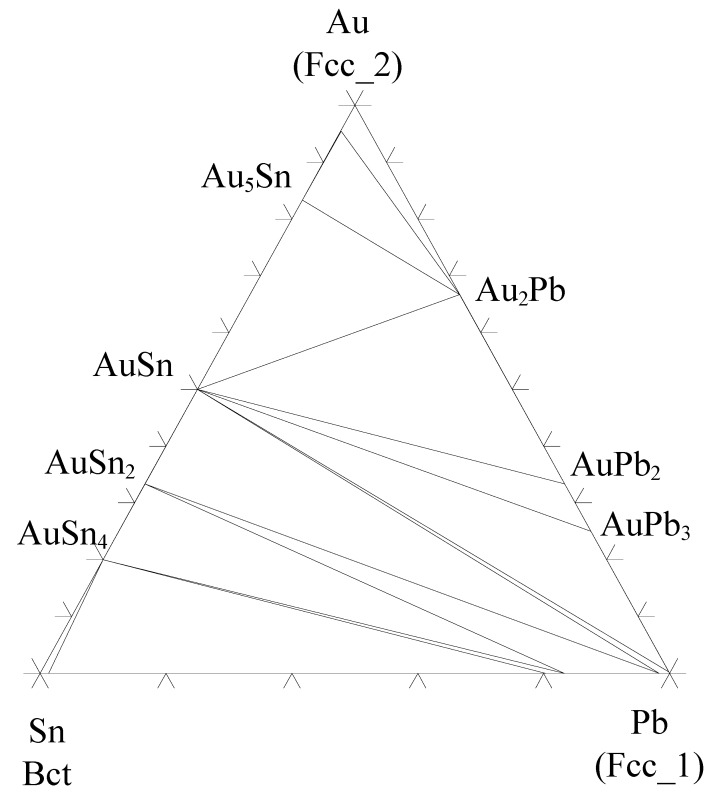
Isothermal section at 150 °C from the evaluated Au-Pb-Sn ternary phase diagram.

The solder side is in all cases either SnPb (Figure 14) or practically pure tin (Figure 18). In the case of almost pure tin the local equilibrium is fulfilled, as (Au,Ni)Sn_4_ can exist in local equilibrium with tin. The more interesting case is the SnPb (or in our case SnPbAg) solder as it can be seen that Pb-rich layer is preferentially located next to (Au,Ni)Sn_4_ layer (Figure 14). From Figure 22 one can see that lead, with about 20 at-% tin dissolved in it, can exist in local equilibrium with AuSn_4_.

Therefore also (Au,Ni)Sn_4_ can most probably exist in local equilibrium with Pb saturated with Sn, thus explaining why the Pb-rich layer can be located beside the (Au,Ni)Sn_4_ layer. The preferential location of Pb-rich layer in contact with (Au,Ni)Sn_4_ has sometimes been explained with the help of interfacial energies [58] as it is known that Pb can form low energy interfaces [59]. Nevertheless, as no reliable experimental values for the surface energies of solids exist, the explanation is highly qualitative. The more probable reason is that tin has been consumed during the reactions with Au and Ni and Pb-rich layer has been left behind. It should be noticed that the layer cannot be pure lead but instead be saturated with Sn as only Pb(Sn) can exist in local equilibrium with AuSn_4_ (and also probably with (Au,Ni)Sn_4_). As lead and gold form intermetallics an appropriate question is, why they are not formed. From the Au-Pb-Sn isothermal section at 150 °C it can be seen that AuPb-intermetallics can exist in local equilibrium only with almost pure lead, not lead that is saturated with tin. But if the Pb-rich layer becomes almost pure lead (as more and more tin is consumed) during the evolution of the microstructure, as annealing is continued, AuPb-intermetallic compounds can start to form. Before this can take place, one should see the formation of two other AuSn intermetallic compounds i.e., AuSn_2_ and AuSn, between AuSn_4_ and lead-rich layer as the tin content of the Pb-rich region decreases. This was not detected after annealing up to 3,000 hours.

**Figure 23:**
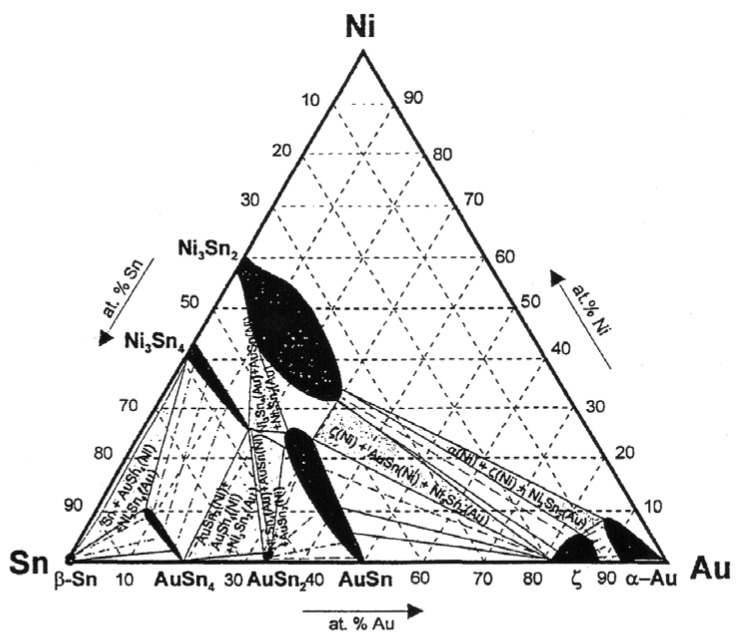
Isothermal section of the Au-Ni-Sn system at room temperature [60] (© 1998 IEEE).

When considering the interface between Ni_3_Sn_4_ and AuSn_4_ (Figure 14 and Figure 18) the situation is quite similar than above. Based on the ternary Au-Ni-Sn phase diagram determined experimentally at room temperature [60] it is obvious that both phases involved in the above-discussed reaction (Ni_3_Sn_4_ and AuSn_4_) can exist in local equilibrium and further that they exhibit extended solid solubilities (Figure 23 and Ref. [61]). Based on that what has been stated above, it is not surprising that (Au,Ni)Sn_4_ redeposits on top of Ni_3_Sn_4._ This is due to the following reasons: firstly, (Au,Ni)Sn_4_ can exist in local equilibrium with Ni_3_Sn_4._ From Figure 23 it can immediately be seen that in order to local equilibrium to be fulfilled at both interfaces [Ni_3_Sn_4_/(Au,Ni)Sn_4_ and (Au,Ni)Sn_4_/Sn (Figure 18)] the (Au,Ni)Sn_4_ must contain the maximum amount of Ni in the Au-sublattice. Under these conditions the Au content of Ni_3_Sn_4_ is likewise restricted to almost zero. This explains why we could not detect any Au inside Ni_3_Sn_4_, since only almost pure Ni_3_Sn_4_, (Au,Ni)Sn_4_ with maximum amount of Ni and Sn fulfill the local equilibrium requirement (Figure 23). Secondly, based on the thermodynamic calculations, we know that Ni has a very strong stabilizing effect on (Cu,Ni)_6_Sn_5_ [62]_._

Thus, it is very likely that Ni also stabilizes (Au,Ni)Sn_4_, since Ni exhibits extensive ternary solubility to (Au,Ni)Sn_4_. This could act as the driving force for the reaction, since the total Gibbs free energy of the system could be lowered in this way. In order to quantify the second reason i.e., the effect of Ni on the stability of (Au,Ni)Sn_4,_ thermodynamic description of the Au-Sn-Ni ternary system should be available. Unfortunately, at the moment this kind of description is unavailable. As Ni_3_Sn_4_ forms already during the melting of the solders (Table 1 and Table 2) the formation of the layered structure occurs most likely as follows: During solid-state annealing Au diffuses towards the Ni_3_Sn_4_ because or the stabilizing effect of Ni on the (Au,Ni)Sn_4_. Au reacts with Ni_3_Sn_4_ to release Ni that is subsequently incorporated to the growing (Au,Ni)Sn_4_. Since (Au,Ni)Sn_4_ must exist in equilibrium at the same time with both Ni_3_Sn_4_ and Sn (Figure 23), it will dissolve the maximum amount of Ni into the Au-sublattice.

**Figure 24:**
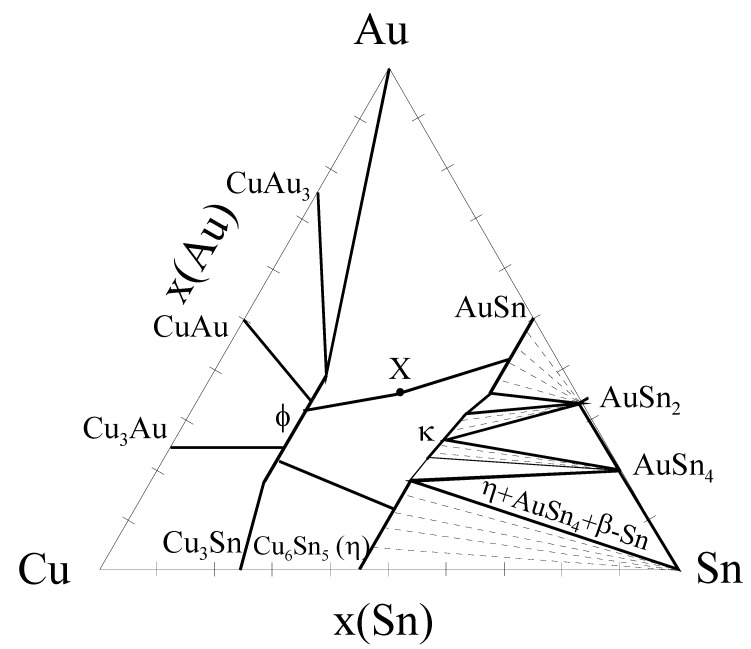
Experimentally determined isothermal section through the Au-Sn-Cu system at 170 °C. Redrawn from [63].

As has been described, Cu-containing solders do not exhibit the redeposition of (Au,Ni)Sn_4_ to the interface. In fact some of the available Au is incorporated into the (Cu,Au,Ni)_6_Sn_5_ compound (Figure 20). Further, no Ni_3_Sn_4_ is formed owing to the reasons discussed above. The formation of (Cu,Au,Ni)_6_Sn_5_ also seems to reduce the total intermetallic reaction rate owing to the limited mass-supply of Cu (Table 3). This result is consistent with the growth rate information available in the literature [21]. It is not immediately clear why redeposition of AuSn_4_ does not occur when Cu is present in the solder. One reason may be that Cu_6_Sn_5_ cannot exist in equilibrium with the AuSn_4_.However, from the experimentally determined partial isothermal section of the Au-Cu-Sn systems at 170 °C (Figure 24) by Roeder [63] it can be seen that Cu_6_Sn_5_ and AuSn_4_ can in fact exist in local equilibrium and this explanation cannot be correct. However, what is different when compared to the local equilibrium established between Ni_3_Sn_4_ and AuSn_4_ is that AuSn_4_ does not dissolve Cu as it does Ni. This implies that Cu should not stabilize AuSn_4_ as much as Ni does. Further, one can see that Cu_6_Sn_5_ can dissolve extensive amounts of Au. In fact, from the diagram one can see that the local equilibrium between AuSn_4_ and (Cu,Au)_6_Sn_5_ (as well as with Sn) is possible only if the amount of Au in (Cu,Au)_6_Sn_5_ is close to 20 at-% (Figure 24). Thus, with small Au layer thickness used in practice the redeposition of AuSn_4_ should not occur, since it is highly unlikely that such high Au concentrations inside (Cu,Au)_6_Sn_5_ could be realized. Nevertheless, in this investigations there should be enough Au available and the above-described equilibrium should be attainable. The appropriate question would then be why AuSn_4_ does not redeposit on the top of (Cu,Ni,Au)_6_Sn_5._ We propose that this is owing to the following reasons: The limited solubility of Cu in AuSn_4_ indicates that Cu does not markedly increase the stability of AuSn_4_. Further, the stabilizing effect of Ni on the (Cu,Ni)_6_Sn_5_ ensures that the Ni is strongly bounded in the Cu-Sn-IMC and is not available for AuSn_4_. The dissolution of Ni (and also probably that of Au) makes the (Cu,Ni,Au)_6_Sn_5_ stable enough that the AuSn_4_ cannot decompose it and form (Au,Ni)Sn_4_. Hence, since there is no Ni available at the IMC/solder interface the AuSn_4_ remains inside the bulk solder. The maximum amount of Au that we found from the (Cu,Au,Ni)_6_Sn_5_ in any of the samples was about 12 at-%. The isothermal section in Figure 23 has been determined at 170 °C, whereas our annealing experiments were carried out at 150 °C. Therefore, the maximum solubility at the annealing temperature should be somewhat smaller than that in Figure 24. Further, since there is also Ni in the (Cu,Au,Ni)_6_Sn_5_ we are actually dealing with quaternary solubility. Thus, the maximum solubility under these conditions may well be the measured 12 at-%. The behavior of Cu_6_Sn_5_ is very interesting since it seems to be able to accommodate various species into its structure. This is expected to be related to the fact that Cu_6_Sn_5_ has NiAs based structure that is known to be very flexible and therefore to be able to incorporate both small and large atoms [64].

The (SnAg)/Ni/Au system is thermodynamically quite similar to that of (SnPbAg)/Ni/Au as already discussed. However, the growth rate is significantly lower in the (SnAg)/Ni/Au system (compare the thickness values in Tables I and II). The (Au,Ni)Sn_4_ follows parabolic growth kinetics throughout the annealing in the (SnPbAg)/Ni/Au system and up to 500 hours of annealing in the (SnAg)/Ni/Au system. When one calculates the parabolic rate constants (k_p_) for the two systems for the annealings up to 500 hours (annealing period where parabolic kinetics dominates in both systems) one ends up to the result: k_p_^SnPbAg^ ≈ 2 × k_p_^SnAg^. The appropriate question is then what causes this difference. It is known that Au can diffuse rapidly in both Sn and Pb phases via (at least partly) interstitial mechanism [65,66]. Results of the Au diffusion in Pb and Sn single crystals show that Au diffusion in lead is slightly faster (about five times, depending on the Sn crystal axis in question) than that of Au in Sn [65,66]. This observation, together with the fact that the bulk of the SnPbAg solder contains much more interfacial area (because of the lamellar eutectic structure), which offer fast interfacial diffusion paths for Au, should lead to higher effective diffusion rate of Au and hence to faster growth rate of (Au,Ni)Sn_4_ in the SnPbAg than in the SnAg system. Thus, the observed thickness decrease of (Au,Ni)Sn_4_ in the SnAg solder case after 1,000 hours of annealing may be related to the gradually decreasing flux of Au from the solder matrix and subsequent mass-supply problems.

## 5. Interfacial Reaction between Zn Containing Solders and Ni/Au Metallization

The relatively high melting temperature of the Sn-Ag-Cu near eutectic solder family restricts their use in certain applications and thus solders with lower melting temperature are needed. Among these Sn-Zn solders offer significant benefits on cost as well as on mechanical properties [25]. However, there are major drawbacks with these alloys that include poor corrosion resistance in humid/high temperature environment and poor compatibility with common substrate materials used in electronics, especially with Ni/Au [67].

**Figure 25:**
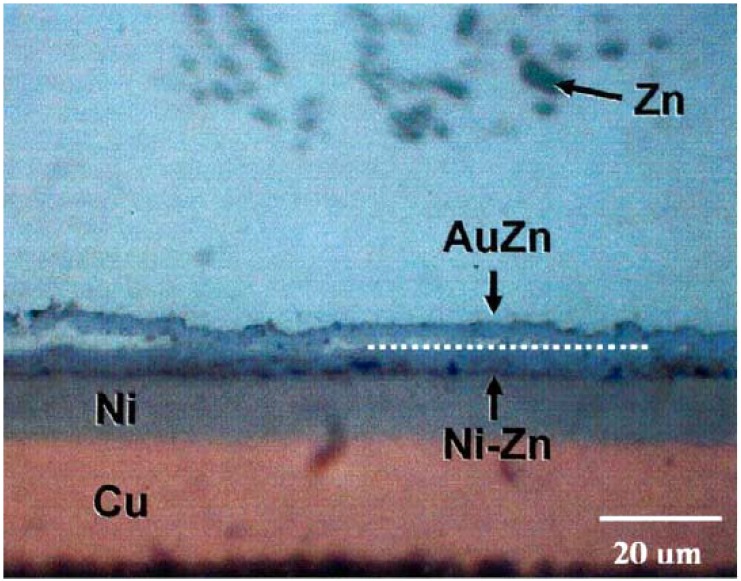
Interfacial microstructure after reflow and 900 hours annealing at 150 ° between Sn-7at-%Zn solder and Au/Ni/Cu pad [68].

**Figure 26:**
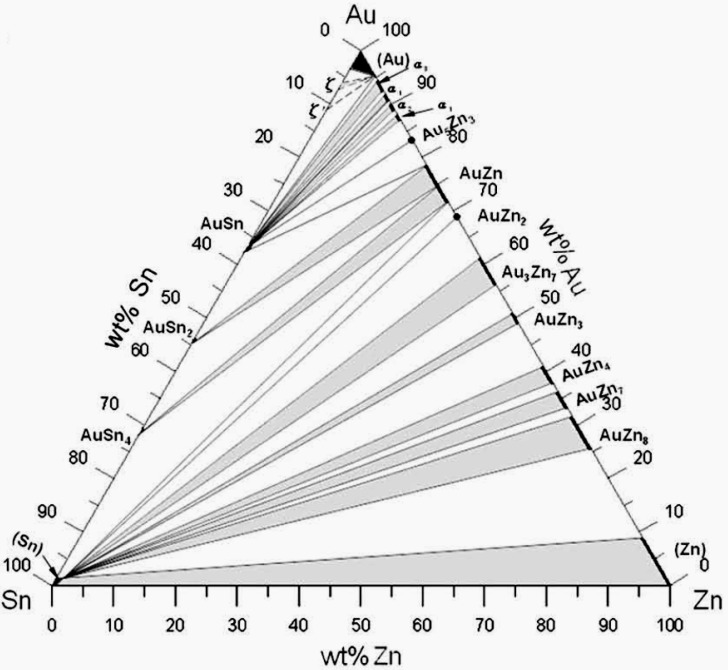
Isothermal section of the Au-Sn-Zn system at 175 °C [69].

Kim *et al.* [68] studied reactions between Sn-7at-%Zn solder and Au/Ni/Cu (0.5–1 μm/10–15 μm/22–32 μm) pad structures. Peak reflow temperature was 240 ± 5 °C and time above liquidus was 60 s. The samples were subsequently annealed at 150 °C for 600, 900 and 1,500 hours. After reflow there was already tendency for the interfacial reaction product layer to peel of from the interface and float into the bulk solder. It is reported in the abstract of the publication that this layer is AuZn, but there is no chemical analysis presented in the publication. After aging for 900 h the interfacial reaction layer structure is as shown in Figure 25. Now, there are two layers at the interface; AuZn (about 5 μm) and Ni_5_Zn_21_ (about 7 μm). It is also evident that the interface between the two layers is not particulary stable mechanically as it seems that some of the AuZn is peeling of the Ni_5_Zn_21_. The authors also found out that the Ni_5_Zn_21_ layer actually consisted of three separate layers with slight differences in composition and lattice parameter. From Figure 25 it is also clearly seen that the number of Zn precipitates is much smaller near the interface than further away. There are two reasons for this: (i) some of the Zn is consumed during the formation of the AuZn layer and (ii) based on the ternary Au-Sn-Zn phase diagram (Figure 26) [69] the local equlibrium at the AuZn/solder interface requires that the Zn content in the solder decreases to about 2.3 at-% (1.3 wt-%). The thickness of the AuZn layer did not increase upon further annealing owing to the limited mass-supply of Au in the interconnection structure. The total thickness of the Ni_5_Zn_21_ grew to about 10 μm after annealing for 1,500 hours.

Lin *et al*. [70] studied the reactions between Sn-9 wt-%Zn (about 15 at-%) and Au/Ni(0.5μm/5μm)/Cu pads. The time above liquidus during the reflow was about 70 s. After reflow the samples were annealed in solid state at 100 °C and 150 °C for various time periods. After reflow the interfacial IMC tended to float away (as in [68]) from the interface. The chemical analyses pointed out that the layer consisted of two parts, one with composition close to AuZn_4_ and other with Au_7_Zn_18_ (γ3 phase in the Au-Zn binary system [71]). After aging at 100 °C the AuZn_4_ layer grows at the expence of Au_7_Zn_18_, which is reasonable, as Au_7_Zn_18_ is not stable at 100 °C and should thus dissappear. Also the Zn content near the floating layer decreases as it should according to the Au-Sn-Zn phase diagram. No further reactions at the solder/Ni interface are observed even with 1,000 h at 100 °C. However, after annealing at 150 °C Ni_4_Zn_21_ phase is observed at the Ni/solder interface. The composition corresponds to the γ-phase in the Ni-Zn phase diagram [72]. The phase in question has a extended solubility range and the composition reported in the present publication is located near the Zn-rich phase boundary. Upon further annealing the Ni_4_Zn_21_ phase grows in thickness. Based on the time vs. thickness results presented in the publication the growth is diffusion controlled. The Zn precipitates also decrease in number near the interfacial reaction structure as the Zn content in the matrix approaches the local equilibrium condition. The authors also present shear strength measurements which show that after reflow the samples have the highest shear strength values (8.6 N, not much difference whether they have been reflowed one, two or three times). After 100 hours of aging the value drops slightly (to about 7.2 N) and then stays constant upon further annealing. There is no difference between samples aged at 100 °C and 150 °C despite the fact that only at 150 °C there is Ni_4_Zn_21_ at the interface. This indicates that the properties of the solder matrix control the fracture behaviour, which is reasonable with low shear rates used in the publication.

Chang *et al*. [27] studied interfacial reactions between Sn-9wt-%Zn and Au/Ni (0.5–0.7 μm/6–8 μm) pads after reflow and subsequent solid state annealing at 175 °C. Again the peeling of the intermetallic reaction layer from the interface was observed already after reflow. The IMC in question consisted of two parts with compositions corresponding to Au_3_Zn_7_ and AuZn_3_. When one compares these results with the results of Kim [68] and Lin [70] it is evident that in this case the Au-Zn compounds are richer in Zn than in the other two cases. This can be rationalized by considering that in [68] the thickness of the Au layer was somewhat higher and in [70] the bumps size was smaller (so the Au content was higher despite the same layer thickness). Upon annealing at 175 °C the Au-Zn layers gradually transformed to AuZn_4_ and then to AuZn_8_ with some Zn next to it, thus realizing the three phase (AuZn_8_–Zn–Sn[Zn]) local equilibrium (see Figure 26). At the Ni/Solder interface Ni_5_Zn_21_ layer formed (the same γ phase as in the other publications with slightly higher Ni content) and it grew up to 20 μm after 50 days of annealing. Again the Zn content of the solder matrix near the IMC layers decreased owing to the reasons discussed above. The authors also observed that the AuZn_4_ IMC inside the bulk solder was actually (Au,Ni)Zn_4_. Thus, it seems that Ni can also stabilize AuZn_4_ like it stablizes AuSn_4_ (see Section 3).

As a summary from the results presented above, it can be stated that typically the reaction between Sn-Zn solder and Au/Ni metallization starts by formation of Au-Zn IMC’s (after dissolution of Au layer) that then float away from the interface to bulk solder. At the Ni/solder interface the formation of γ-Ni-Zn phase takes place when the temperature is higher than 100 °C. Depending on the amount of Au in the system the final Au-Zn IMC inside the solder matrix varies. The γ-Ni-Zn phase grows by diffusion controlled kinetics and its thickness can reach relatively high values (especially at 175 °C). Thus, based on this information the interfacial properties of solder interconnection with Sn-Zn solder and Au/Ni metallization are most probably problematic from the reliability point of view, especially under high deformation rates (shock impact) where the interfacial IMC’s control the fracture behaviour of the solder joints.

## 6. Interfacial Reactions between SnBi Solders and Ni Metallization

Eutectic SnBi solder is often used when low soldering temperatures are needed. The fact that Ni reacts with both Sn and Bi can induce some complexity to the situation when compared to SnBi solders used with Cu metallization. There are few investigations concerning interfacial reactions with eutectic SnBi solders and Ni metallization as well as phase diagram information concerning the Sn-Bi-Ni system [54,72,73,74,75].

**Figure 27:**
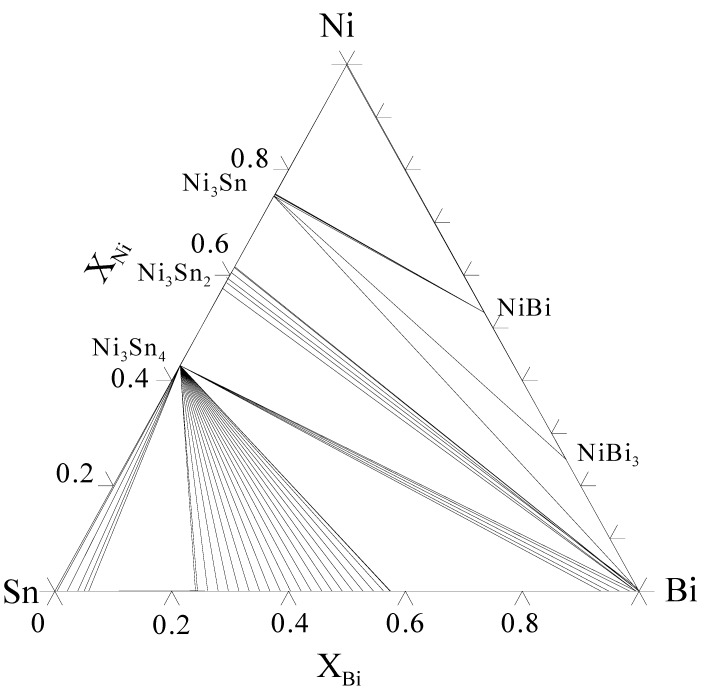
Isothermal section of the Bi-Ni-Sn system at 180 °C.

The study carried out by Chen *et al*. [72] showed that when Ni metallization reacted with SnBi eutectic solder only Ni_3_Sn_4_ was formed at the interface. Even after solid state annealing at 135 °C for 3,600 °C, no other phases were observable. Also the thickness of the Ni_3_Sn_4_ was small (16 µm) thus indicating slow growth kinetics typical for Ni-Sn IMC’s. There was a relatively thick Bi-rich layer adjacent to the Ni_3_Sn_4_ layer, which was formed due to the consumption of Sn from the solder during the formation of the Ni_3_Sn_4_. Despite this, no Ni-Bi IMC were observed. The reason for this can be rationalized with the help of isothermal section from the Bi-Ni-Sn system calculated at 180 °C (Figure 27) from the assessed thermodynamic data [54]. From the diagram it is evident that in order to NiBi_3_ to become into local equilibrium at the SnBi-solder/Ni interface the Sn content of the Bi-rich band should be practically zero. Thus, the situation is very similar to that in the Au-Pb-Sn system [24]. It is anticipated that this situation can occur only when the annealing times are very long or if the solder volume is extremely small (i.e., so called small volume effect [76]).

## 7. Summary and Conclusions

In this paper we have shown how thermodynamic-kinetic method can be utilized to rationalize a wide range of interfacial phenomena between Sn-based lead-free solders and Ni metallizations. First, effect of P on the interfacial reactions, and thus on the reliability, between Sn-based solders (both Pb-free and Pb-containing) and ENIG metallizations, was rationalized. By utilizing available thermodynamic data and kinetic considerations, a reaction model was proposed. In addition, the variation in the P content of the Ni(P) metallization was discussed and analyzed. Next, the effect of small amounts of Cu in Sn-based solders on the intermetallic compound (IMC), which forms first on top of Ni metallization was discussed. With the help of thermodynamic arguments a so called critical Cu concentration for the formation of (Cu,Ni)_6_Sn_5_ was determined as a function of temperature. The effect of additional alloying elements, such as Ag and Bi, present in the solder was analyzed. As (Cu,Ni)_6_Sn_5_ has been observed to produce relaibility problems during drop testing, it is important to understand the phenomena behind the formation of the interfacial reaction layers on top of Ni, when Cu is present in the system. It is to be noted that Cu can also enter the soldering system from, for example, component metallization, and thus has not be present initially inside the solder to have the effects described in the paper. The effect of minor amounts of Cu on the IMC layer formed at the Ni/solder interface makes also comparison of results obtained with Pb-free solder to those with SnPb solder slightly problematic. Then the important phenomenon of redeposition of (Au,Ni)Sn_4_ layer on top of Ni_3_Sn_4_ IMC was discussed in detail. The reasons leading to this behaviour were rationalized with the help of thermodynamic information and the explanation why this phenomenon does not occur when appropriate amount of Cu is present in the soldering system was given. In addition, the difference in the kinetics of the redeposition between SnPbAg and SnAg solders was discussed by utilizing the available kinetic information. Next, interfacial reaction issues realted to Sn-Zn based solders and Ni metallization were analyzed with available thermodynamic information. Based on the discussion reliability problems under shock loading can be expected if Sn-Zn solders are used with Ni metallization. Finally, reaction between another low melting point solder (eutectic SnBi) and Ni were analysed with the help of assessed thermodynamic information. The reasons for the absence of NiBi IMC layers was explained by utilizing the isothermal section (at appropriate temperature) from the Sn-Bi-Ni system. Hence, as a conclusion it can be stated that the present method can be used to analyse a wide range of interfacial phenomena in electronics and other fields of technology and science. The only restriction is the amount of data available to be used in the calculations.
